# Alisertib Induces Cell Cycle Arrest, Apoptosis, Autophagy and Suppresses EMT in HT29 and Caco-2 Cells

**DOI:** 10.3390/ijms17010041

**Published:** 2015-12-29

**Authors:** Bao-Jun Ren, Zhi-Wei Zhou, Da-Jian Zhu, Yong-Le Ju, Jin-Hao Wu, Man-Zhao Ouyang, Xiao-Wu Chen, Shu-Feng Zhou

**Affiliations:** 1 Department of Gastrointestinal Surgery, Shunde First People’s Hospital Affiliated to Southern Medical University, Guangdong 528300, China; rbjsdyy@outlook.com (B.-J.R.); zdjsdyy@outlook.com (D.-J.Z.); jylsdyy@outlook.com (Y.-L.J.); wjhsdyy@outlook.com (J.-H.W.); oymzsdyy@outlook.com (M.-Z.O.); 2 Department of Pharmaceutical Sciences, College of Pharmacy, University of South Florida, 12901 Bruce B. Downs Blvd., MDC 30, Tampa, FL 33612, USA; zzhou1@health.usf.edu

**Keywords:** alisertib, colorectal cancer, cell cycle, programmed cell death, EMT

## Abstract

Colorectal cancer (CRC) is one of the most common malignancies worldwide with substantial mortality and morbidity. Alisertib (ALS) is a selective Aurora kinase A (AURKA) inhibitor with unclear effect and molecular interactome on CRC. This study aimed to evaluate the molecular interactome and anticancer effect of ALS and explore the underlying mechanisms in HT29 and Caco-2 cells. ALS markedly arrested cells in G_2_/M phase in both cell lines, accompanied by remarkable alterations in the expression level of key cell cycle regulators. ALS induced apoptosis in HT29 and Caco-2 cells through mitochondrial and death receptor pathways. ALS also induced autophagy in HT29 and Caco-2 cells, with the suppression of phosphoinositide 3-kinase (PI3K)/protein kinase B (Akt)/mammalian target of rapamycin (mTOR), but activation of 5′ AMP-activated protein kinase (AMPK) signaling pathways. There was a differential modulating effect of ALS on p38 MAPK signaling pathway in both cell lines. Moreover, induction or inhibition of autophagy modulated basal and ALS-induced apoptosis in both cell lines. ALS potently suppressed epithelial to mesenchymal transition (EMT) in HT29 and Caco-2 cells. Collectively, it suggests that induction of cell cycle arrest, promotion of apoptosis and autophagy, and suppression of EMT involving mitochondrial, death receptor, PI3K/Akt/mTOR, p38 MAPK, and AMPK signaling pathways contribute to the cancer cell killing effect of ALS on CRC cells.

## 1. Introduction

Colorectal cancer (CRC) is the third most common malignancy in male and the second most common one in female worldwide in 2012 [1]. It was estimated that about 136,830 new cases and 50,310 deaths occurred in US which renders CRC as the third leading cause of cancer related death in 2014 [2]. In China, CRC was the fifth most common cancer and the fifth leading cause of cancer death in 2011 [3]. There were 253,000 new CRC cases, 139,000 CRC deaths, and 583,000 people living with CRC (within 5 years of diagnosis) in 2012 in China [1]. The overall 5-year survival rate is up to 90% in patients with localized CRC [4]; however, it was only 5%–8% in patients with distant metastases in USA [5]. In clinic, there were about 20%–30% of patients who were newly diagnosed with unresectable metastatic disease and 50%–60% patients with CRC will develop metastatic disease, mainly due to the epithelial to mesenchymal transition (EMT) [6]. Therefore, it is of great importance to develop new therapeutics for CRC treatment, in particular, the distant metastatic CRC.

In addition to the primary surgical resection, the current systemic chemotherapies for CRC, including FOLFIRI (5-fluorouracil, leucovorin and irinotecan) and FOLFOX (5-fluorouracil, leucovorin and oxaliplatin) are the cornerstone of treatment for metastatic CRC patients. The advent of targeted agents (e.g., the anti-vascular endothelial growth factor monoclonal antibody bevacizumab or the anti-epidermal growth factor receptor monoclonal antibodies cetuximab and panitumumab) were recognized as a landmark advance in the treatment of metastatic CRC. However, in many patients, all of the above schemes lead to a poor outcome due to the drug-related adverse events and drug resistance [7]. Thus, it is of clinical importance to identify more effective biological agents with specific therapeutic targets and reduced side effect.

There were three members of Aurora kinases (Aurora A, B, and C) that are affiliated to a family of serine/threonine kinases in mammals, and Aurora kinases have emerged as crucial mitotic regulators required for genome stability [8]. The Aurora kinases regulate multiple aspects of mitosis, including centrosome duplication, spindle assembly, chromosome alignment, chromosome segregation and the fidelity-monitoring spindle checkpoint [9]. Aurora kinase A (AURKA) localizes to duplicated centrosomes and spindle poles, with key functions of centrosomes maturation, timing of mitotic entry and construction and control of a bipolar spindle. Aberrant mRNA or protein expression of AURKA causes abnormalities in mitosis (e.g., aneuploidy, supernumerary centrosomes, and defective mitotic spindles) and induces resistance to apoptosis. In clinic, amplification or overexpression of AURKA was observed in many solid cancers, such as ovarian, gastric, pancreatic, and breast cancers [10]. Also, the abnormal condition of AURKA has been frequently investigated in CRC and is associated with poor prognosis and response to chemotherapy [11,12]. The expression of AURKA protein is higher in CRC liver metastasis than the corresponding primary tumor, which can be recognized as a molecular biomarker with prognostic value for patients with CRC liver metastasis, independent of established clinicopathological variables [13]. It was also suggested that AURKA was essential for CRC stem cells regeneration and resistance to cytotoxic stimuli in CRC [14].

Given the specific localization and function in cells and pathological activation of AURKA in many malignant diseases, AURKA have been an attractive target for the development of new therapeutic approaches for cancer treatment. Numerous compounds of AURKA inhibitor have undergone preclinical testing into Phase I or II trials, including AMG900, AT9283, MLN8237, AZD1152, and ENMD2076 [15]. MLN8237, also known as alisertib (ALS, Figure S1A), is one of secondary generation AURKA inhibitors. *In vitro*, ALS has displayed its effects on inhibition of proliferation and cell cycle progression in glioblastoma neurosphere tumor, malignant bladder cancer, pancreatic cancer, ovarian cancer, breast cancer, osteosarcoma, gastric cancer, and esophageal adenocarcinoma cell lines [16,17,18,19,20,21,22]. The anticancer effect of ALS was also examined *in vivo* in multiple myeloma and acute lymphoblastic leukemia xenograft models [23]. Implanted tumors shrunk considerably in multiple myeloma models and the overall survival or disease-free survival was significantly improved in animal models.

However, the role of AURKA in the tumorigenesis and development of CRC and the underlying mechanism have not been fully elucidated, which renders the anticancer effect and molecular mechanisms of ALS in the treatment of CRC remain unclear. In this study, we aimed to unveil the molecular targets, examine the cancer cell killing effect of ALS and elucidate the molecular mechanism for its anticancer effect, with a focus on the cell proliferation, cell cycle distribution, programmed cell death, and EMT in human CRC cell lines HT29 and Caco-2 cells.

## 2. Results

### 2.1. Alisertib (ALS) Inhibits the Proliferation of HT29 and Caco-2 Cells

We first examined the effect of ALS on the viability of HT29 and Caco-2 cells using 3-(4,5-dimethylthiazol-2-yl)-2,5-diphenyltetrazolium bromide (MTT) assay. Treatment of both cell lines with ALS at concentrations ranging from 0.1 to 100 μM for 24 or 48 h significantly decreased the viability (Figure S1B,C). Compared with the control cells, the viability of HT29 cells was decreased from 78.5% to 47.3% when exposed to ALS for 24 h and declined from 71.0% to 31.2% when treated with ALS for 48 h at concentrations from 0.1 to 100 μM, respectively (Figure S1B). The *IC*_50_ values were 49.31 and 17.86 μM for HT29 cells after 24 and 48 h incubation with ALS, respectively. As shown in Figure S1C, the percentage of the viability of Caco-2 cells was decreased from 89.7% to 45.7% when treated with ALS for 24 h and declined from 100.3% to 18.8% when incubated with ALS for 48 h at concentrations from 0.1 to 100 μM, respectively. The *IC*_50_ values were 88.8 and 52.1 μM for Caco-2 cells after 24 and 48 h incubation with ALS, respectively. These results suggest that ALS inhibits the cell proliferation in concentration- and time-dependent manners and displays a potent inhibitory effect on the growth of HT29 and Caco-2 cells.

### 2.2. Overview of Proteomic Response to ALS Treatment in HT29 and Caco-2 Cells

Following, we performed a stable isotope labeling by amino acids in cell culture (SILAC)-based proteomic study to quantitatively determine the molecular interactome in HT-29 and Caco-2 cells responding to ALS. There were 893 and 582 protein molecules identified as the potential targets of ALS in HT-29 (Table 1) and Caco-2 cells (Table 2), respectively. ALS increased the expression level of 459 protein molecules, while decreasing the expression level of 434 protein molecules in HT-29 cells. In Caco-2 cells, ALS increased the expression level of 207 protein molecules, but decreased the expression level of 375 protein molecules. Subsequently, these proteins were subject to IPA pathway and network analysis. There were 24 and 25 networks of signaling pathway and cellular function that were potentially regulated by ALS in HT-29 and Caco-2 cells, respectively. These included a number of molecules involved in cell proliferation, metabolism, migration, invasion, survival, and death (Table 1 and Table 2). As such, the following experiments were performed to validate the effect of ALS in HT-29 and Caco-2 cells, with a focus on cell cycle distribution, programmed cell death, and EMT.

**Table 1:** Molecules and networks were regulated by alisertib (ALS) in HT-29 cells.

ID	Molecules in Network	Score	Focus Molecules	Top Diseases and Functions
1	60S ribosomal ubunit, DDX1, DDX5, DDX17, DDX21, DDX3X, DHX9, EIF3E, HDGF, HNRNPU, ILF2, ILF3, KPNA6, LYAR, MTDH, NEDD8, RAD23B, RBM39, Rnr, RPL3, RPL5, RPL8, RPL9, RPL10, RPL14, RPL18, RPL24, RPL28, RPL30, RPL27A, RPLP0, RPS2, RPS15A, Vegf, XRCC5	52	32	Protein synthesis, infectious disease, gene expression
2	AHSA1, ALYREF, CAND1, ERK1/2, FUBP1, GIGYF2, HNRNPK, HNRNPL, LARS, LOC102724594/U2AF1, MARS, NOLC1, NOP56, NOP58, OLA1, PABPC1, PCBP1, PCBP2, PDE6H, Pki, PTBP1, PUF60, QARS, RALY, RNA polymerase I, SF3B1, SF3B2, Srp30, SRSF1, SRSF2, SRSF3, Tap, TOP1, U2af, U2AF2	44	29	RNA post-transcriptional modification, protein synthesis, cancer
3	CALU, CLIC4, creatine kinase, CUTA, EFTUD2, FUS, Histone H1, HNRNPC, HNRNPD, HNRNPF, HNRNPH1, HNRNPM, HNRNPUL1, Importin α, Importin β, IPO5, IPO7, P38 MAPK, PQBP1, PRMT1, PRMT5, PRPF19, PTMA, PYCR1, RAN, RANBP1, SF3B4, snRNP, SNRPD3, SYNC, TNPO1, Transportin, TRAP/Media, UBA2, WDR1	40	27	RNA post-transcriptional modification, amino acid metabolism, post-translational modification
4	AGR2, Akt, ANXA4, Calcineurin A, CALR, Collagen α1, Collagen type VI, COP I, COPA, COPB1, COPG1, Cytoplasmic Dynein, DSTN, DYNC1LI2, FKBP4, Mre11, PA2G4, PDIA3, PDIA4, PDIA6, peptidylprolyl isomerase, peroxidase (miscellaneous), PPIA, PPIB, PPT1, PRDX1, PRDX2, PRDX4, PRDX5, PRDX6, RRBP1, SAE1, TMED9, TXNDC5, ZYX	38	26	Cancer, endocrine system disorders, organismal injury and abnormalities
5	ACTN1, ACTN4, ANP32B, ASPH, ATP synthase, C1QBP, CAMK2D, CaMKII, COX5A, CSRP1, CTNNA1, Cytochrome bc1, cytochrome C, ERP44, Filamin, ITPR, KLK3, LONP1, MAP4, Mitochondrial complex 1, MRPL12, MT-CO1, MT-CO2, MT-CYB, NDUFA5, Pde, PDS5A, PDXDC1, PHB, PHB2, Pka, PPP2R1A, PRDX3, PYGB, STMN1	38	26	Drug metabolism, lipid metabolism, small molecule biochemistry
6	19S proteasome, 20s proteasome, ADRM1, BUB3, COTL1, CPNE1, DAD1, FLII, Immunoproteasome Pa28/20s, MHC CLASS I (family), NFkB (complex), OTUB1, Proteasome PA700/20s, PSMA, PSMA4, PSMA7, PSMB5, PSMC2, PSMD, PSMD2, PSMD6, PSMD8, PSMD14, PSME1, PSME2, RNF13, S100, S100A2, S100P, UBA1, UBE2, UBE2L3, UBE2N, USMG5, USP14	35	25	Cellular movement, protein degradation, protein synthesis
7	adenosine-tetraphosphatase, α tubulin, ATP5H, ATP5J, ATP5J2, β Tubulin, BZW1, BZW2, CCT4, CCT5, CCT8, CCT6A, DCTN1, Dynein, Ephb, ERP29, F0 ATP synthase, FAM162A, H2AFV, KDELR, LGALS3BP, MAPK1, OGFR, PGAM1, SAR1A, Sec23, TBCA, TSPO, TUBA4A, TUBB6, tubulin (family), VBP1, Vdac, VDAC1, VDAC2	35	25	Cellular assembly and organization, cell-to-cell signaling and interaction, reproductive system development and function
8	CD9, chymotrypsin, CTSH, EIF1, EIF3, EIF3A, EIF3B, EIF3F, EIF3G, EIF3J, EIF3L, EIF4B, Eif4g, Erm, EZR, G6PD, GLRX3, GPIIB-IIIA, IPO9, Lamin b, LBR, Lh, PEBP1, Pkc(s), Pkg, PLCB3, PLIN3, PSIP1, RAB1A, RBM3, Rho gdi, SERPINB1, SLC12A2, TLN1, VAT1	35	25	Gene expression, protein synthesis, cellular growth and proliferation
9	ABCB8, Aconitase, Adaptor protein 1, Aldose Reductase, Angiotensin II receptor type 1, AP1B1, Arf, ARF1, ARF4, ARF5, ARL1, COPB2, CRIP2, cytochrome-c oxidase, DAB2IP, DHCR24, Gef, glutathione peroxidase, glutathione transferase, GST, GSTK1, GSTO1, GSTP1, Jnk, MGST1, PARK7, RAB1B, RPN2, SFXN1, SLC25A3, SLC25A11, SOD2, SRP54, SSR3, USO1	33	24	Drug metabolism, protein synthesis, DNA replication, recombination, and repair
10	ABCC1, AK2, ALDH2, ANXA3, ATPase, atypical protein kinase C, CAPG, Caveolin, CLTA, DBNL, DNPEP, Dynamin, ETFA, ETFB, GANAB, IL-2R, Integrin α 3 β 1, MTCH2, NSF, NUDC, OSBP, PAK2, PCMT1, PHGDH, PI3K (complex), Ptk, Raf, RAP1B, SEPT11, SHMT2, SLC3A2, TPP2, trypsin, VAPA, Vla-4	33	24	Developmental disorder, hereditary disorder, metabolic disease
11	ALDOA, AP2B1, C11orf54, CAD, CBX3, CYP, DHCR7, DPP3, Focal adhesion kinase, GGCT, Histone h4, HMGCS1, IDI1, Ldh (complex), LDHA, LDHB, MAT2A, NPC2, NQO1, PI3K (family), POR, Rar, RBBP4, RBP2, RPN1, Rxr, SLC16A1, Sod, Sos, STAT5a/b, TGM2, thymidine kinase, TPP1, TXNRD1, UGDH	33	24	DNA replication, recombination, and repair, energy production, nucleic acid metabolism
12	ACAA1, APEX1, BTF3, BTF3L4, Cbp/p300, CPSF6, Cyclin A, Cyclin D, Cyclin E, E2f, EDF1, EEF2, EPCAM, FEN1, Hat, HAT1, Holo RNA polymerase II, HSD17B4, Ku, Mcm, MCM3, MCM7, MTHFD1, NASP, NONO, PCNA, POLβ-POLepsilon-POLγ-XRCC1-LIGI-PARP1-PCNA-FEN1, POLR2H, PRKDC, RAD50, Ras, Rb, SSRP1, TIP60, XRCC6	29	22	DNA replication, recombination, and repair, cellular response to therapeutics, cell morphology
13	ANXA5, BCR (complex), CAPZA2, CAPZB, caspase, CDC37, DENR, EIF4G1, FAS, FLNA, FLNB, Hsp27, HSPB1, IFIH1, JINK1/2, Lamin, LMNB1, MAP2K1/2, MCTS1, Mlc, MTORC2, MTPN, Pak, PARP, PARP1, PDCD6, PLEC, PP2A, PYGL, RHOA, Rsk, RTN3, RTN4, Sapk, SRP72	29	22	Cellular compromise, cell morphology, cellular movement
14	ACTG1, ATP5C1, Calmodulin, Caspase 3/7, CD3, CD44, CSTF2, DDX19A, ECH1, EEF1D, Eif2, HNRNPR, HSPA5, Ifn γ, LMNA, MYH9, PGK1, PPM1G, Proinsulin, RAB10, RAB8A, Ribosomal 40s subunit, RPS7, RPS24, RPS3A, RPSA, Secretase γ, SRPRB, TCF, TCR, Tgf β, TSTA3, TUBB, tubulin (complex), tyrosine kinase	29	22	Hematological disease, immunological disease, inflammatory disease
15	26s Proteasome, AMPK, CD59, CS, CTBP1, CTBP2, DDB1, GFPT1, HDL, hemoglobin, HISTONE, Hsp70, Hsp90, HUWE1, IDH3A, IL1, LMAN1, MIF, NADPH oxidase, Nos, NSUN2, PRKAA, Pro-inflammatory Cytokine, PTGES3, Ras homolog, SDHA, SGTA, SSBP1, succinate dehydrogenase, SURF4, TMED2, TRAP1, Ubiquitin, UBXN1, UCHL3	25	20	Cellular assembly and organization, cellular function and maintenance, cellular development
16	ABCE1, ACLY, Ap1, ATP1A1, Calcineurin protein(s), CK1, Ck2, Gsk3, HMBS, HMGA1, HMGB1, HMGB2, HMGB3, HNRNPAB, MEF2, Mek, MVP, NAP1L1, NFAT (complex), Nfat (family), NMDA Receptor, NUDT5, p70 S6k, PDAP1, Pdgf (complex), phosphatase, PICALM, PP1 protein complex group, PP1-C, PPP1R7, Ppp2c, STARD10, TECR, WARS, XPO1	24	19	DNA replication, recombination, and repair, gene expression, nucleic acid metabolism
17	ACTB, Actin, ACTR2, aldo, α actin, α Actinin, α catenin, API5, Arp2/3, ARPC2, ARPC3, Cadherin, CAP1, CLIC1, CNN2, Cofilin, DIAPH1, DPYSL2, ERK, F Actin, FCGR1A/2A/3A, G-Actin, GOT1, LASP1, LTA4H, MYH14, Profilin, Rock, Talin, TPM3, TPM4, Tropomyosin, Troponin t, TWF1, VASP	22	18	Cellular assembly and organization, cellular function and maintenance, cell morphology
18	Adaptor protein 2, AHR, AKR1A1, APP, ATIC, BIN3, BOLA2/BOLA2B, C11orf54, C14orf93, Clathrin, CORO1C, CPN2, CUL3, DDX55, DNAAF2, ERH, EWSR1, FAM98B, GCSH, HEATR5A, MYC, NLE1, NUDT21, OARD1, OLFML2A, PAICS, RAB7A, SLC25A1, SRSF10, TKT, TMEM183A, TPGS2, UBC, UBL4A, UGT1A9 (includes others)	22	19	Cell cycle, hepatic system development and function, cell morphology
19	ACY1, APMAP, APRT, BBS7, BCKDK, C21orf33/LOC102724023, CARHSP1, CLN5, CMPK1, CNN3, CNPY2, CRYZ, CS, EFHD2, FAM98B, GLS, IARS2, KIF20A, MARCH8, MDH1, MTAP, MYLIP, NME3, NME4, NME7, PADI2, PGM3, RAB6B, RABGAP1, RASA4, REXO4, SCT, SLC25A22, TTC1, UBC	20	20	Nucleic acid metabolism, small molecule biochemistry, cell-to-cell signaling and interaction
20	ACSL3, ADCY, ADRB, Alp, ANP32E, ANXA2, Creb, CTNNB1, DPY30, estrogen receptor, G protein, G protein α, G protein β γ, GTPase, Hdac, Histone h3, HSPA9, Insulin, MATR3, Mmp, NOMO1 (includes others), p85 (pik3r), Pdgfr, PLC, PMM2, RCC2, RNA polymerase II, SFPQ, Shc, SRC (family), STIP1, SUMO2, TOP2A, WDR12, WDR36	18	16	Cell cycle, hair and skin development and function, cancer
21	APPBP2, ARIH2, ARL4D, CD70, CDV3, COQ6, EDNRA, ELF4, EML4, GALE, GMDS, H32, HKDC1, ISOC2, KIF6, LSM8, MAGEA11, NME4, NME1-NME2, NUP210L, OARD1, PCSK5, PODXL, RBM47, RRS1, SCFD1, SELL, SYT11, TBL3, TCIRG1, TNKS, TRMT1, UBC, YBX2, ZCCHC12	15	15	Cell-to-cell signaling and interaction, hematological system development and function, immune cell trafficking
22	AAMP, ANXA1, calpain, Casein, Collagen type I, Collagen type III, Collagen type IV, Collagen(s), COMT, Cpla2, DDAH1, Fc γ receptor, Fibrin, Fibrinogen, GADD45, Growth hormone, Integrin, Laminin, LAMP1, LDL, Mac1, Mapk, MCFD2, NAMPT, PDGF BB, PLCE1, Pld, Rap1, SEC13, SERPINH1, SLIRP, SYK, thyroid hormone receptor, TSH, VAV	12	12	Lipid metabolism, molecular transport, small molecule biochemistry
23	AChR, ALDOC, FABP5, Fcer1, Gm-csf, GOT, HINT1, HLA-DR, HSP, Ifn, IFN β, Ige, IgG, IgG1, IgG2a, Igm, Ikb, Ikk (family), IL12 (complex), IL12 (family), Immunoglobulin, Interferon α, mediator, MHC Class I (complex), MHC Class II (complex), NACA, NPEPPS, OGDH, PI3K p85, PLA2, PLC γ, PRKRA, Rac, Tlr, TXLNA	7	8	Cancer, hematological disease, immunological disease
24	ACPP, ACTR2, ARHGAP1, CBR1, CBR3, CDC42, CDC42EP4, Cg, chemokine, CLDN11, COL15A1, collagen, DHCR7, Endothelin, EPHA3, FCGR1A/2A/3A, FSH, GNRH, GNRH2, GNRHR, HSD3B1, Hsd3b4 (includes others), IKK (complex), LIMK2, Metalloprotease, MTORC1, MYO5B, NPC1L1, PAPPA, PEPD, PHKA2, RAB11A, Tnf (family), TNFRSF6B, UCN2	4	8	Endocrine system development and function, lipid metabolism, small molecule biochemistry

**Table 2:** Molecules and networks were regulated by ALS in Caco-2 cell.

ID	Molecules in Network	Score	Focus Molecules	Top Diseases and Functions
1	60S ribosomal subunit, AARS, AHSA1, AIMP1, C11orf58, DARS, EEF1A1, EEF1B2, EEF1D, EEF1G, EPRS, ERK1/2, GARS, HARS, KARS, MARS, OLA1, PDE6H, PDGF (family), Pki, QARS, RPL8, RPL10, RPL18, RPL21, RPL23, RPL27, RPL30, RPL32, RPL10A, RPL13A, RPLP0, RPLP1, RPS3A, VARS	48	31	Protein synthesis, gene expression, RNA post-transcriptional modification
2	ANXA3, APEX1, BTF3, Cbp/p300, CDH17, CTNNB1, CYB5R3, ESD, FEN1, GANAB, GOLPH3, GST, GSTO1, HDLBP, HIST2H2AC, Holo RNA polymerase II, LMNA, NACA, OAT, PCNA, PGAM1, PRKCSH, PRKDC, RUVBL1, SERBP1, SLC38A2, SSRP1, SUPT16H, TAGLN2, TCF/LEF, thymidine kinase, TMPO, XRCC5, XRCC6, YBX3	45	30	DNA replication, recombination, and repair, cellular response to therapeutics, cell morphology
3	DDOST, DDX17, FUBP1, FUS, hnRNP H, HNRNPA1, HNRNPDL, HNRNPF, HNRNPH1, HNRNPK, HNRNPL, HNRNPR, HNRNPU, IGF2BP3, Karyopherin β, KHSRP, Mapk, MATR3, NONO, PCBP1, PCBP2, PDGF-AA, PSPC1, PTBP1, PUF60, RAN, RAN-GTP, RANGAP1, RBM14, SF3A3, SFPQ, SYNCRIP, TNPO1, Transportin, YBX1	43	29	RNA post-transcriptional modification, protein synthesis, DNA replication, recombination, and repair
4	14-3-3 (β, ε, ζ), CALU, CAPZA1, CAPZB, caspase, CLTC, DLAT, FLNA, GNB2L1, HMBS, HNRNPM, Hsp27, Hsp90, HSP90AA1, HSP90AB1, HSPA8, NCL, NPM1, NUDT21, NUMA1, p85 (pik3r), PCMT1, PKM, PLEC, RPL12, RPL22, SFMBT2, SPTBN1, TRIM28, tubulin (complex), UBXN1, VPS35, YWHAB, YWHAE, YWHAH	43	29	Cancer, gastrointestinal disease, hepatic system disease
5	14-3-3(β, γ, θ, η, ζ), AChR, ACTR3, ALDOC, ALYREF, ATP5A1, Calmodulin, DDX39B, DYNC1H1, EPCAM, F1 ATPase, FABP5, GTPase, IARS2, IMMT, IQGAP1, KIF5B, LRPPRC, MARCKS, mediator, MYH9, Pde, RPL3, RPL4, RPL7, RPLP2, RTN4, SARNP, SLC25A3, TPD52L2, TPI1, TUFM, YWHAG, YWHAQ, ZFC3H1	40	28	Metabolic disease, molecular transport, RNA trafficking
6	14-3-3(η, θ, ζ), Cytokeratin, DSP, EIF3, EIF2S2, EIF3A, EIF3B, EIF3C, EIF3E, EIF3F, EIF3I, EIF3M, EIF4A, EIF4A1, EIF4A3, EIF4B, EIF4F, Eif4g, EIF4G1, EIF4G2, EIF4H, GPI, KRT1, KRT2, KRT8, KRT9, KRT14, KRT18, KRT19, p70 S6k, PABPC1, PI3K (complex), PKP2, PNN, YWHAZ	38	27	Gene expression, protein synthesis, cellular assembly and organization
7	3-hydroxyacyl-CoA dehydrogenase, ACAA1, ACAA2, ACAT1, ACAT2, acetyl-CoA C-acetyltransferase, acetyl-CoA C-acyltransferase, CD99, CNBP, DDX21, DHX9, EDC4, FBL, GLUD1, GSR, H2AFY, HADH, HADHA, HDGF, HNRNPA3, HSD17B10, LOC102724594/U2AF1, NFkB (complex), OTUB1, PDLIM1, peptidase, PPARα-RXRα, RBM39, RCC2, SRSF1, Tap, TOP1, U2af, U2AF2, UBE2	36	26	Renal damage, renal tubule injury, endocrine system development and function
8	Adaptor protein 1, α tubulin, Ap1 γ, AP1B1, AP1G1, β Tubulin, CCT4, CCT5, CCT8, CCT6A, CNN3, CS, DPYSL2, Dynein, EHD1, ERP29, ETFB, FH, Integrin α 5 β 1, LCP1, malate dehydrogenase, MAPK1, MDH1, MDH2, TKT, TUBA1B, TUBB6, TUBB8, TUBB2B, TUBB4B, tubulin (family), Vdac, VDAC1, VDAC2, WARS	36	26	Cancer, hematological disease, immunological disease
9	adenosine-tetraphosphatase, ATP synthase, ATP5B, ATP5H, collagen, Collagen α1, Collagen type III, Cytoplasmic Dynein, DCTN2, DHX15, EFTUD2, FKBP4, HNMT, HNRNPA2B1, MAPRE1, P38 MAPK, PA2G4, peroxidase (miscellaneous), PGK1, PPIA, PRDX1, PRDX2, PRDX6, PRPF8, PRPF19, RRBP1, SERPINH1, SF3B1, SF3B2, SND1, snRNP, SNRPD1, STIP1, TARS, VIL1	36	26	RNA post-transcriptional modification, free radical scavenging, small molecule biochemistry
10	ACLY, ALDH1A1, ANXA5, CK1, CYB5B, DHCR7, DNAJA2, ECHS1, FASN, Focal adhesion kinase, HMGCS1, HSP, Hsp70, HSPA4, HSPA9, HSPB1, HSPE1, HSPH1, IDI1, JINK1/2, MHC Class II (complex), PGD, Pias, POR, PPA1, PSAP, SLC25A6, Srebp, ST13, TOMM22, TOMM40, UBA1, UBE2L3, Ubiquitin, UGDH	36	26	Cell cycle, endocrine system development and function, lipid metabolism
11	ADK, Akt, ARHGDIA, atypical protein kinase C, CADM1, CAPRIN1, CPNE1, EEF2, Fascin, GDI2, HN1, ILF2, ILF3, LRRC47, Mcm, MCM2, MCM3, MCM4, MCM6, N-Cadherin, Pak, PLIN3, PPP2R1B, Rab5, Rab11, RAB11A, RAB2A, RAB7A, RDX, Rho gdi, RNH1, RPA, SEC13, TWF2, VAT1	34	25	DNA replication, recombination, and repair, cell signaling, post-translational modification
12	ADH5, API5, Arp2/3, ARPC2, ARPC5, CLIC1, CNN2, DDX1, Eif2, ERK, GPIIB-IIIA, HNRNPH3, IGF2BP1, Ku, LAMB1, Laminin1, LAP3, Profilin, Ribosomal 40s subunit, Rnr, RPS2, RPS7, RPS8, RPS10, RPS12, RPS14, RPS15, RPS24, RPS27A, RPS4X, RPSA, RTCB, Talin, TLN1, VCL	34	25	Developmental disorder, hematological disease, hereditary disorder
13	Arf, ARF1, ARHGAP1, B2M, B2m-Mhc1a, CALR, CANX, CCAR2, CCDC47, Cd1, CD1D-CANX-CALR-ERp57, COP I, COPA, COPB1, COPE, COPG1, DNAJ, DNAJA1, DNAJC8, HLA-B27, Hsp22/Hsp40/Hsp90, HSP90B1, HSPA5, HYOU1, Jnk, LMAN1, MHC Class I (complex), P4HB, PDIA3, PDIA4, PDIA6, PRDX4, RAB1B, RPN1, TXNDC5	34	25	Post-translational modification, protein folding, developmental disorder
14	ACO2, Aconitase, AHCY, AKR1C3, AKR1C1/AKR1C2, Aldose Reductase, CACYBP, chymotrypsin, COMT, ENO1, ERP44, ETF1, Filamin, FLNB, FLNC, G6PD, GADD45, GCN1L1, glutathione peroxidase, Lamin b, LONP1, LRRC59, ME1, N-cor, PARK7, PEBP1, PFKP, Pkc(s), PTGES3, Rar, RPL7A, SEPT2, T3-TR-RXR, TALDO1, thyroid hormone receptor	30	23	Nucleic acid metabolism, small molecule biochemistry, cellular movement
15	14-3-3, APC/APC2, ATIC, CBX3, CHD4, CLIC4, CSE1L, GART, H3F3A/H3F3B, HIST1H1C, Histone H1, Histone h3, Histone h4, IDH1, IL-2R, Importin α, Importin β, IPO5, IPO7, KPNA2, KPNA3, KPNB1, Mucin, NuRD, NUTF2, PAICS, PHGDH, PTMA, RANBP1, SAE1, SNRPD3, SSB, TIP60, Vegf, WDR1	30	23	Molecular transport, protein trafficking, amino acid metabolism
16	ACTA1, ACTG1, Actin, ACTN4, ACTR2, aldo, α actin, α Actinin, BASP1, Cadherin, CAP1, CFL1, CFL2, CKB, Cofilin, CORO1C, COTL1, DBN1, F Actin, G-Actin, LMO7, MYH10, MYL12A, Myosin2, NAPA, NWASP, PAFAH1B2, PDCD6IP, Pka, PLS3, TMOD3, TPM3, TPM4, Tropomyosin, TWF1	30	23	Cellular assembly and organization, cellular function and maintenance, developmental disorder
17	19S proteasome, 20s proteasome, 26s Proteasome, ATP5C1, ATPase, CCT3, CCT7, Cyclin E, Immunoproteasome Pa28/20s, LRP, MHC CLASS I (family), NSFL1C, Proteasome PA700/20s, PSMA, PSMA1, PSMA2, PSMA4, PSMA7, PSMB1, PSMC4, PSMD, PSMD1, PSMD2, PSMD3, PSME1, PSME2, Rac, SLC3A2, SOD1, SQSTM1, TUBB, UBE2N, USP5, VAPA, VCP	30	23	Developmental disorder, hereditary disorder, inflammatory disease
18	ACTN1, α catenin, ANXA2, ATP6V1A, C1QBP, CAPNS1, Caveolin, CDHE/CDHN, CSRP1, CTNNA1, CTNND1, CTNNα-CTNNβ-CTNNδ, CYC1, Cyclin B, Cytochrome bc1, cytochrome C, cytochrome-c oxidase, DNM1L, Dynamin, EPPK1, ETFA, HSPD1, Il8r, JUP, MDK, Mitochondrial complex 1, PARP, PHB, PHB2, PRDX3, Ras homolog, SPTAN1, STOML2, UQCRC1, ZYX	28	22	Cellular movement, cell morphology, cellular function and maintenance
19	ALDOA, AP2B1, APOA1, ATP2A2, calpain, CAPN1, CAPN2, Casein, creatine kinase, DBI, DPP3, Fibrinogen, GC, Growth hormone, HBD, HDL, hemoglobin, HIST1H2BL, Ldh (complex), LDHA, LDHB, LDL, NAMPT, Nos, Nr1h, PF4, Pro-inflammatory Cytokine, QDPR, SAA, SERPINA1, Sod, SRC (family), TFRC, TGM2, VLDL-cholesterol	22	19	Cellular function and maintenance, carbohydrate metabolism, free radical scavenging
20	Alp, APLP2, BCAT1, CDH1, Collagen type I, Collagen type IV, Collagen(s), CTSB, CTTN, elastase, F11R, Fgf, Fgfr, Fibrin, FN1, GLG1, GOT, GOT1, GOT2, GPD2, Integrin, Laminin, LMNB1, MAT2A, Mmp, Notch, PDGF BB, Rap1, RPN2, Secretase γ, SERPINA3, SERPINB1, TAGLN, Tgf β, trypsin	19	17	Amino acid metabolism, small molecule biochemistry, dermatological diseases and conditions
21	ACTL8, AGPAT2, ANP32E, BROX, CDV3, CEP78, CHMP6, CHMP7, CHMP1B, CHMP2A, CHMP4A, CHMP4B, CMPK1, GMPS, HEBP1, HIST1H2AE, HSDL2, NAP1L2, NAP1L4, NARS, NME3, NME4, NME7, NME1-NME2, NPM3, PNPO, QPRT, RNPEP, TCEAL1, TSN, TTLL4, TUBAL3, UBC, USP54, ZBTB18	17	16	Infectious disease, cell morphology, cellular assembly and organization
22	ATP1A1, ATP6V1B2, Calcineurin A, CCT2, Ck2, Dishevelled, EIF2S1, EIF5A, Gsk3, ITPR, MAP2K1/2, MIR124, Mlc, Pdgf (complex), PFN1, phosphatase, PICALM, Pkg, PP1 protein complex group, PP1-C, PP1/PP2A, PP2A, PPP1CA, Ppp2c, PPP2R4, PPP2R1A, PRKAA, Rb, RCN1, Rock, SET, SHMT2, Spectrin, STARD10, XPO1	16	15	Molecular transport, RNA trafficking, hereditary disorder
23	ACKR1, ACSL3, AFP, ALDH18A1, AP4S1, APLNR, CCND1, CKAP4, DPY30, E2f, EMR2, EMR3, ENOPH1, FPR3, FSD1, γ tubulin, Gpcr, GPR15, GPR35, GPR137B, GPRC5C, Metalloprotease, MFSD1, miR-491-5p (and other miRNAs w/seed GUGGGGA), NAP1L1, NDC1, NUP155, PXN, RAB10, RXFP2, SSR4, STAT, STX2, TTLL4, UBC	9	10	Cell-to-cell signaling and interaction, cellular assembly and organization, cellular compromise
24	AHNAK, BCR (complex), Complement component 1, CXADR, DDX6, ENaC, EZR, Fcer1, GTF2I, HINT1, HLA-DR, Iga, Ige, IgG1, Igg3, Igm, Ikb, IMPDH2, JAK, KHDRBS1, MEF2, MIRLET7, NFAT (complex), Nfat (family), NFkB (family), PI3K (family), PI3K p85, PLC γ, Ptk, Raf, Ras, Rsk, Sapk, SYK/ZAP, TEC	7	9	Cancer, organismal injury and abnormalities, infectious disease
25	Adaptor protein 2, ADCY, ADRB, Ap1, BSG, Calcineurin protein(s), CaMKII, CDK1, Cg, Clathrin, Creb, DDX5, estrogen receptor, FSH, G protein, GFPT1, GML, GSTP1, IKK (complex), Insulin, Lh, MCTS1, MTORC1, NADPH oxidase, NMDA Receptor, Pdgfr, Pka catalytic subunit, PLC, Proinsulin, RNA polymerase II, Shc, SKP1, Sos, TCF, UCHL1	7	9	Neurological disease, psychological disorders, skeletal and muscular disorders

### 2.3. ALS Decreases the Phosphorylation Level of Aurora Kinase A (AURKA) in HT29 and Caco-2 Cells

**Figure 1:**
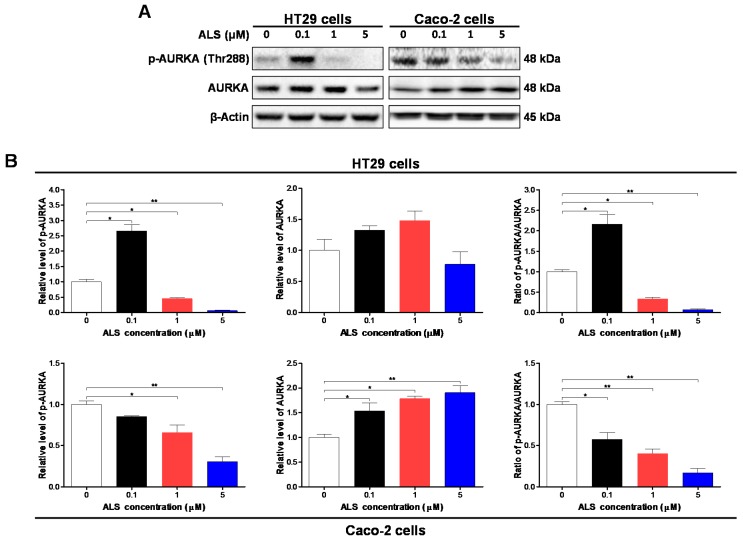
Alisertib (ALS) inhibits the phosphorylation of Aurora kinase A (AURKA) in HT29 and Caco-2 cells. HT29 and Caco-2 cells were exposed to ALS at 0.1, 1, and 5 μM for 48 h and protein samples were subject to Western blotting assay. (**A**) Representative blots of p-AURKA and total AURKA examined by Western blotting assay; (**B**) Bar graphs showing the level of p-AURKA and AURKA in HT29 and Caco-2 cells. β-Actin was used as the internal control. Data are shown as the mean ± SD of three independent experiments. * *p* < 0.05 and ** *p* < 0.01 by one-way analysis of variance (ANOVA).

To determine whether ALS effectively targeted AURKA and affected cellular mitosis, we first examined the phosphorylation level of AURKA and the total expression level of AURKA after HT29 and Caco-2 cells were treated with ALS at 0.1, 1, and 5 μM for 48 h. Intriguingly, there were variable alterations in the phosphorylation and expression level of AURKA in these two cell lines when exposed to ALS (Figure 1A,B). In HT29 cells, the phosphorylation level of AURKA was decreased 54.4% and 93.7% when treated with ALS at 1 and 5 μM for 48 h, respectively (*p* < 0.001; Figure 1A,B). However, there was no significant difference in the expression level of AURKA (*p* > 0.05). Consequently, it led to a 66.4% and 93% reduction in the ratio of p-AURKA/AURKA when HT29 cells were treated with ALS 1 and 5 μM for 48 h, respectively, (*p* < 0.05; Figure 1A,B).

Also, as shown in Figure 1, treatment of Caco-2 cells with ALS significantly inhibited the phosphorylation of AURKA at Thr288 in a concentration-dependent manner, whereas there was no significant change in the expression level of AURKA when treated with ALS at 0.1, 1, and 5 μM for 48 h. Moreover, in comparison to the control cells, incubation of Caco-2 cells with ALS at 0.1, 1, and 5 μM led to a 42.4%, 59.5%, and 82.9% reduction in the ratio of p-AURKA over AURKA, respectively (*p* < 0.05; Figure 1A,B). Collectively, treatment of HT29 and Caco-2 cells with ALS significantly inhibits the phosphorylation of AURKA at Thr288 in a concentration-dependent manner.

### 2.4. ALS Modulates the Cell Cycle Distribution of HT29 and Caco-2 Cells

**Figure 2:**
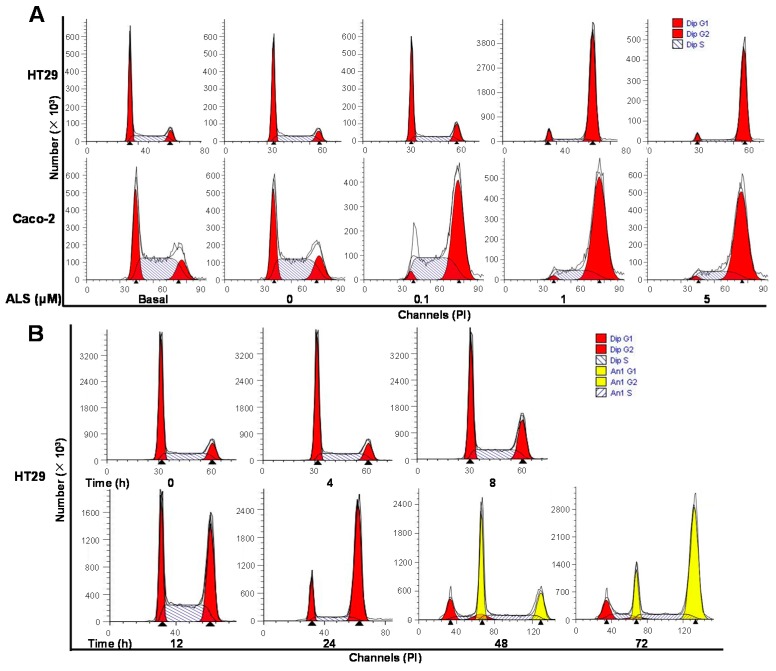

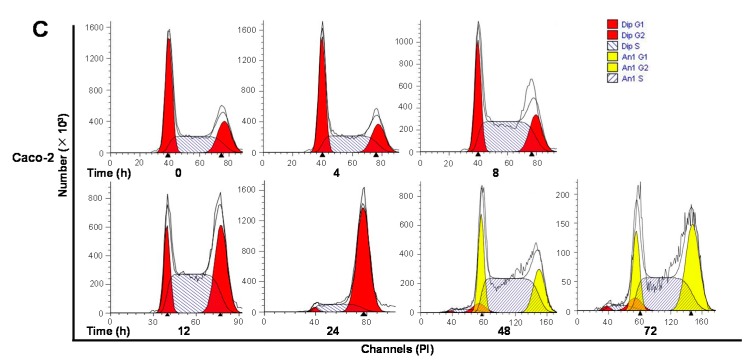
ALS induces cell cycle arrest in G2/M phase in HT29 and Caco-2 cells. (**A**) HT29 and Caco-2 cells were treated with ALS at 0.1, 1, and 5 μM for 24 h and then subjected to flow cytometric analysis. Representative DNA fluorescence histograms of propidium iodide (PI)-stained HT29 and Caco-2 cells showing the cell cycle distribution; (**B**) Time course of ALS-induced cell cycle change over 72 h in HT29 cells. Flow cytometric histograms showing the cell cycle distribution when HT29 cells were incubated with ALS at 1 μM for 4, 8, 12, 24, 48, and 72 h; (**C**) Time course of ALS-induced cell cycle change over 72 h in Caco-2 cells. Flow cytometric histograms show the cell cycle distribution when Caco-2 cells were incubated with ALS at 1 μM for 4, 8, 12, 24, 48, and 72 h. Cells were stained with PI and subjected to flow cytometric analysis that collected 15,000 events. Data represent the mean ± SD of three independent experiments. Dip: diploid; Ani: aneuploid.

As the inhibitory effect of ALS on cell proliferation and phosphorylation of AURKA has been observed, we next assessed the effect of ALS on the cell cycle distribution of HT29 and Caco-2 cells by flow cytometry. Treatment of HT29 cells with ALS at 0.1, 1, and 5 μM for 24 h resulted in a remarkable increase in the percentage of cells in G_2_/M phase from 10.5% at basal level to 16.8%, 85.7%, and 87.7%, respectively (*p* < 0.001; Figure 2A and Figure S2A). Similarly, the percentage of Caco-2 cells in G_2_/M phase was raised from 17.3% at basal level to 56.2%, 77.2%, and 77.5% when treated with ALS at 0.1, 1, and 5 μM for 24 h, respectively (*p* < 0.001; Figure 2A and Figure S2A). Meanwhile, the percentage of both cell lines in the G_1_ and S phases was decreased correspondingly. The data show that ALS induces significant cell cycle arrest in the G_2_/M phase in both cell lines in a concentration-dependent manner.

We also performed separate experiments to assess the effect of ALS at 1 μM on cell cycle distribution over 72 h in HT29 and Caco-2 cells. We found the appearance of aneuploid cells after ALS treatment over 48 h in both cell lines. Compared with control cells, the percentage of diploid HT29 cells in G_2_/M phase was increased from 7.0% at the basal level to 12.7%, 24.3%, 37.3%, and 71.4% after treatment with 1 μM ALS for 4, 8, 12, and 24 h and declined to 21.1% and 12% after 48 and 72 h, respectively (*p* < 0.001; Figure 2B and Figure S2B), while the percentage of aneuploid HT29 cells in G_2_/M phase was increased from 23.9% to 69.3% after treatment with ALS from 48 to 72 h (Figure 2B and Figure S2B). Similarly, the percentage of diploid Caco-2 cells in G_2_/M phase increased from 18.5% at the basal level to 19.9%, 20.8%, 34.1%, 80.8%, 64.7% and 57.7% when the cells were incubated with 1 μM ALS for 4, 8, 12, 24, 48 and 72 h, respectively (*p* < 0.001; Figure 2C and Figure S2C), while the percentage of aneuploid Caco-2 cells in G_2_/M phase was increased from 16.5% to 34.2% after treatment with ALS from 48 to 72 h. On the contrary, the percentage of HT29 and Caco-2 diploid cells in G_1_ phase was markedly reduced (*p* < 0.001; Figure 2 and Figure S2). Taken together, these results suggest that ALS induces a time-dependent cell cycle arrest in G_2_/M phase in HT29 and Caco-2 cells.

### 2.5. ALS Differentially Alters Key Regulators of Cell Cycle in HT29 and Caco-2 Cells

**Figure 3:**
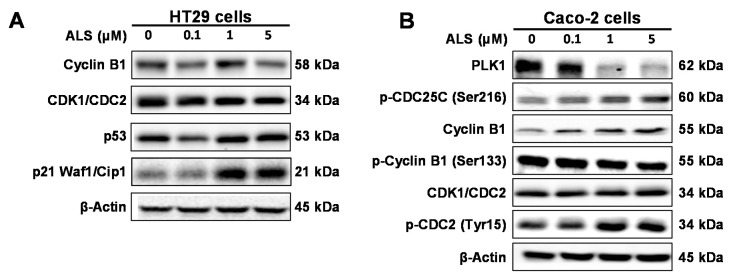
Effect of ALS on the expression level of key regulators of cell cycle in HT29 and Caco-2 cells. (**A**) Representative blots showing the expression of CDK1/CDC2, cyclin B1, p21 Waf1/Cip1, and p53 when HT29 cells were treated with ALS at 0.1, 1, and 5 μM for 48 h; (**B**) Representative blots showing the level of PLK1, CDK1/CDC2, p-CDC2 (Tyr15), cyclin B1, p-cyclin B1 (Ser133), and p-CDC25C (Ser216) in Caco-2 cells.

To unveil the underlying mechanism for ALS-induced cell cycle arrest, we further dissected the expression of key cell cycle regulating proteins, including CDK1/CDC2, p-CDC2 (Tyr15), cyclin B1, p-cyclin B1 (Ser133), p-CDC25C (Ser216), PLK1, p53, and p21/Waf1 in HT29 and Caco-2 cells using Western blotting assay. As shown in Figure 3A and Figure S3A, incubation of HT29 cells with ALS significantly inhibited the expression of the positive regulators but enhanced the expression of negative regulators that are responsible for the G_2_ to M phase transition. The expression level of cyclin B1 and CDC2 was declined in a concentration-dependent manner. There was 1.3-fold reduction and a 30.0% decrease in the expression level of cyclin B1 and CDC2 when treated with 1 μM ALS, respectively (*p* > 0.05), whereas there was a 69.8% and 45.2% reduction in the expression level of cyclin B1 and CDC2 when treated with 5 μM ALS, compared to the control cells, respectively (*p* < 0.05 or 0.01; Figure 3A and Figure S3A). In contrast, the expression level of the negative regulators of cell cycle progression were significantly increased in a concentration-dependent manner. As shown in Figure 3A and Figure S3A, the expression level of p53 was increased 1.5- and 1.4-fold when HT29 cells were incubated with ALS at 1 μM (*p* < 0.05) and 5 μM (*p* > 0.05), respectively, compared to the control cells. The expression level of p21 Waf1/Cip1 was increased 3.3- and 2.5-fold when HT29 cells were treated with 1 and 5 μM ALS, respectively, compared to the control cells (*p* < 0.001; Figure 3A and Figure S3A). Collectively, the decreased expression of cyclin B1 and CDC2 and the increased expression of p53 and p21 contribute to ALS-induced G_2_/M phase arrest of HT29 cells.

For Caco-2 cells, the regulatory effect of ALS on the expression level of key cell cycle regulators was in a concentration-dependent manner (Figure 3B and Figure S3B). We found that the expression level of cyclin B1 and CDC2 were remarkably increased (Figure 3B and Figure S3B). Therefore, we further examined the level of PLK1, p-cyclin B1 (Ser133), p-CDC2 (Tyr15), and p-CDC25C (Ser216). In comparison to the control cells, the level of p-cyclin B1 (Ser133) was decreased 36.2% and 66.4% when treated with 1 and 5 μM ALS for 48 h, respectively (*p <* 0.01; Figure 3B and Figure S3B). Similarly, the expression level of PLK1 was decreased 87.3% and 88.3% when treated with 1 and 5 μM ALS for 48 h, respectively (*p <* 0.01; Figure 3B and Figure S3B). In contrast, the level of p-CDC25C (Ser216) was increased 2.3- and 2.6-fold when cells were incubated with 1 and 5 μM ALS, respectively, compared to the control cells (*p <* 0.01; Figure 3B and Figure S3B). The level of p-CDC2 (Tyr15) was increased 2.8- and 2.7-fold when incubated with 1 and 5 μM ALS, respectively, compared to the control cells (*p* < 0.01; Figure 3B and Figure S3B). Taken together, the decreased level of p-cyclin B1 (Ser133) and PLK1 and the increased level of p-CDC2 (Tyr15), critical regulators for the G_2_/M transition, contribute to ALS-induced G_2_/M phase arrest of Caco-2 cells.

### 2.6. ALS Differentially Induces Cell Death in HT29 and Caco-2 Cells

**Figure 4:**
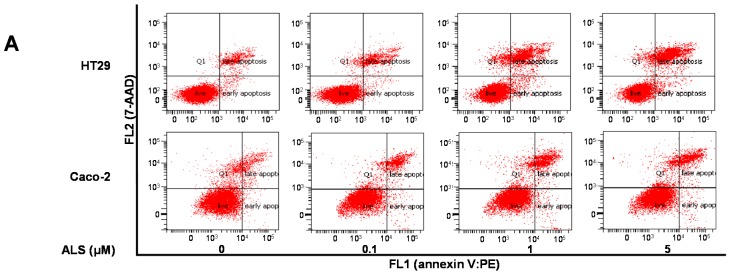

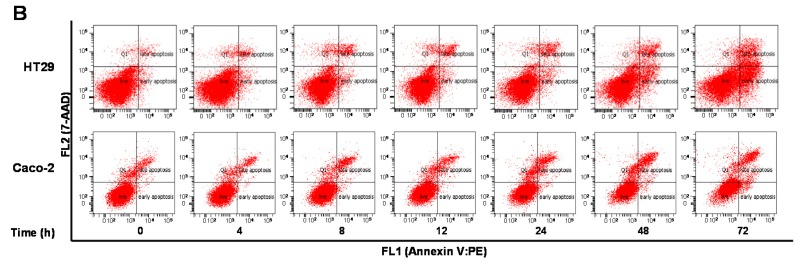
ALS induces apoptotic death in HT29 and Caco-2 cells. (**A**) HT29 and Caco-2 cells were exposed to ALS at 0.1, 1, and 5 μM for 24 h and then subject to flow cytometric analysis. Flow cytometric dot plots of specific cell populations (live, early apoptosis and late apoptosis) in HT29 and Caco-2 cells; (**B**) HT29 and Caco-2 cells were treated with ALS at 1 μM for 4, 8, 12, 24, 48, and 72 h and then subject to flow cytometric analysis. Flow cytometric dot plots showing specific cell populations (live, early apoptosis, and late apoptosis) in HT29 and Caco-2 cells. The apoptotic cells were detected by an annexin-V:PE (phycoerythrin) and 7-Aminoactinomycin D (7-AAD) double staining assay. The flow cytometer collected 15,000 events. Q1 indicated necrotic cells due to the machanical damage. Early apoptotic cells are located in the lower right corner (annexin V:PE positive only). Late apoptotic cells are located in the upper right corner (double positive with annexin V:PE and 7-AAD). The viable cells are located in the lower left corner (double negative with annexin V:PE and 7-AAD staining).

To examine the cancer cell killing effect of ALS on HT29 and Caco-2 cells, the number of apoptotic cells was first quantified using flow cytometry. As shown in Figure 4A and Figure S4A, the total percentage of apoptotic cells (early + late apoptosis) was 6.2% in HT29 cells when incubated with the control vehicle only (0.05% dimethyl sulfoxide (DMSO), *v*/*v*). In comparison with the control cells, there was 2.4- and 2.6-fold increase in total apoptotic cells when HT29 cells were treated with 1 and 5 μM ALS, respectively (*p* < 0.05; Figure 4A and Figure S4A). In Caco-2 cells, there was a 2.0- and 1.8-fold increase in total apoptotic cells when exposed to 1 and 5 μM ALS, compared with the control cells (8.8%), respectively (*p* < 0.05; Figure 4A and Figure S4A).

Next, we evaluated the effect of ALS on the apoptosis of HT29 and Caco-2 cells when cells were exposed to 1 μM ALS over 72 h (Figure 4B and Figure S4B). The percentage of apoptotic HT29 cells was increased from 3.8% at the basal level (time 0) to 4.6%, 4.6%, 5.3%, 6.8%, 12.1%, and 24.3% with the treatment of ALS for 4, 8, 12, 24, 48, and 72 h, respectively. Compared with the control cells, incubation with ALS for 24, 48, and 72 h caused a 1.8-, 3.2-, and 6.4-fold increase in total apoptotic cells (*p* < 0.001; Figure 4B and Figure S4B). For Caco-2 cells, the percentage of apoptotic cells was increased from 6.0% at the basal level (time 0) to 5.9%, 8.7%, 7.6%, 9.4%, 11.8%, and 12.9% with the treatment of ALS for 4, 8, 12, 24, 48, and 72 h, respectively. In comparison to the control cells, treatment of Caco-2 cells with ALS for 24, 48, and 72 h caused a 1.6-, 2.0-, and 2.2-fold increase in total apoptotic cells (*p* < 0.001; Figure 4B and Figure S4B). Taken together, the results clearly suggest that ALS induces apoptotic cell death in HT29 and Caco-2 cells in concentration- and time-dependent manners.

**Figure 5:**
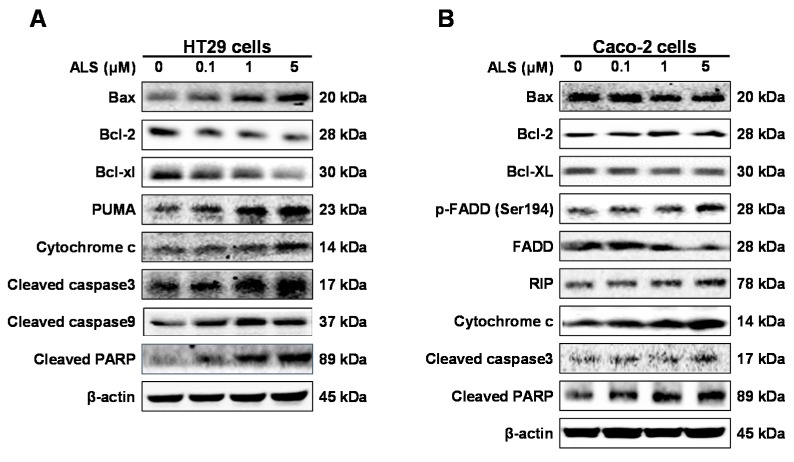
Effect of ALS on the expression level of key proapoptotic and antiapoptotic molecules in HT29 and Caco-2 cells. (**A**) Effect of ALS on the expression level of Bcl-xl, Bax, Bcl-2, PUMA, cytochrome c, cleaved caspase 3, cleaved caspase 9, and cleaved PARP in HT29 cells; (**B**) Effect of ALS on the expression level of Bcl-xl, Bax, Bcl-2, p-FADD (Ser194), FADD, RIP, cytochrome c, cleaved caspase 3, and cleaved PARP in Caco-2 cells.

The mechanism for ALS-induced apoptosis was delineated in HT29 and Caco-2 cells by western blotting analysis. We evaluated the expression level of key molecules involved in apoptosis process, including Bcl-xl, Bcl-2, Bax, PUMA, cytochrome c, cleaved caspase 3, cleaved caspase 9, cleaved PARP, RIP, p-FADD, and FADD. HT29 and Caco-2 cells were treated with ALS at 0.1, 1, and 5 μM for 48 h. In comparison to the control cells, incubation of HT29 cells with 1 and 5 μM ALS increased Bax level 1.8- and 2.1-fold, respectively (*p* < 0.05; Figure 5A and Figure S5A); whereas incubation of HT29 cells with 5 μM ALS decreased Bcl-2 and Bcl-xl level 72.1% and 47.7%, respectively, compared to the control cells (*p* < 0.01 or 0.05; Figure 5A and Figure S5A). Given the marked increase in the expression level of Bax but a dramatic decrease in the expression level of Bcl-xl and Bcl-2, the disturbed balance of mitochondria-mediated apoptotic status may shift to pro-apoptotic state. We assessed the expression of other pro-apoptotic and anti-apoptotic proteins involved in mitochondria-mediated apoptotic pathway. Compared to the control cells, treatment of HT29 cells with 1 and 5 μM ALS for 48 h markedly increased 2.7- and 3.3-fold in the expression level of PUMA, respectively (*p* < 0.01; Figure 5A and Figure S5A). The cytosolic level of cytochrome c was increased 2.6-fold when treated with 5 μM ALS (*p* < 0.05; Figure 5A and Figure S5A). Furthermore, a significant increase in the level of cleaved caspases 9, cleaved caspase 3, and cleaved PARP was observed. Compared to the control cells, incubation of HT29 cells with ALS at 1 and 5 μM resulted in a 2.2- and 2.3-fold increase in the level of cleaved caspase 3, a 2.0- and 1.7-fold rise in the level of cleaved caspase 9, and a 4.4- and 5.3-fold elevation in the level of cleaved PARP, respectively (*p* < 0.01; Figure 5A and Figure S5A). Taken together, these data demonstrate that ALS induces apoptotic death of HT29 cells via mitochondria-mediated apoptotic pathway.

For Caco-2 cells, we also first evaluated the expression levels of the pro-apoptotic protein Bax and the anti-apoptotic proteins Bcl-2 and Bcl-xl. Caco-2 cells were exposed to ALS at 0.1, 1, and 5 μM for 48 h. Surprisingly, the expression level of Bax was decreased 61% and 62.7% when treated with 1 and 5 μM ALS for 48 h, compared with the control cells, respectively (*p* < 0.05; Figure 5B and Figure S5B). In contrast, the level of Bcl-2 was increased 1.5- and 1.5-fold when treated with 1 and 5 μM ALS for 48 h, respectively, compared to the control cells (Figure 5B and Figure S5B). There was no significant alteration in expression level of Bcl-xl between the control cells and ALS-treated cells (Figure 5B and Figure S5B). These results suggest that mitochondrial pathway is not involved in the ALS-induced apoptosis in Caco-2 cells. Therefore, we next assessed the expression level of key proteins regulating death receptor signaling pathway. There was a 1.5- and 2.5-fold increase in the expression level of RIP when cells were treated with ALS at 1 and 5 μM for 48 h, respectively, compared to the control cells (*p* < 0.01; Figure 5B and Figure S5B). There was a 1.5-, 1.3-, and 1.8-fold increase in the level of p-FADD, although there was no statistical significance, while there was a 15.4%, 34.2%, and 36.9% decrease in the expression level of FADD, when cells were incubated in ALS at 0.1, 1, and 5 μM for 48 h, respectively, compared to the control cells (Figure 5B and Figure S5B). Consequently, the p-FADD/FADD ratio was remarkably increased 2.0- and 3.0-fold when cells were treated with 1 and 5 μM ALS for 48 h, respectively, compared to control cells (*p* < 0.01; Figure 5B and Figure S5B). Furthermore, the expression of downstream regulators was also tested. There was a 2.3- and 3.2-fold increase in the cytosolic level of cytochrome c when cells were treated with ALS at 1 and 5 μM for 48 h, respectively, compared to the control cells (*p* < 0.05; Figure 5B and Figure S5B). There was a 1.9- and 2.2-fold increase in the level of cleaved caspase 3 when treated with ALS at 1 and 5 μM for 48 h, respectively, compared to the control cells (*p* < 0.01; Figure 5B and Figure S5B). There was a 1.9- and 2.1-fold increase in the level of cleaved PARP when treated with ALS at 1 and 5 μM for 48 h, respectively, compared to the control cells (*p* < 0.01; Figure 5B and Figure S5B). Taken together, these results reveal that ALS induces apoptotic death of Caco-2 cells via death receptor signaling pathway. The differential responses to ALS treatment may be ascribed to the different cell type, in particular, the difference in p53 in these cell lines.

### 2.7. ALS Induces Autophagy of HT29 and Caco-2 Cells

**Figure 6:**
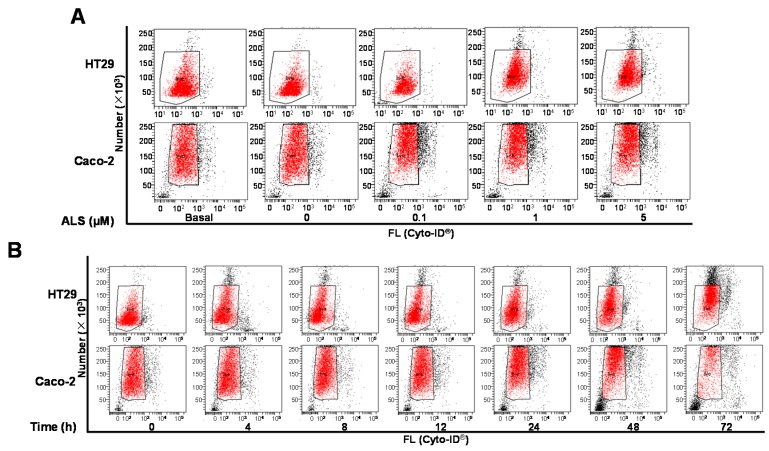

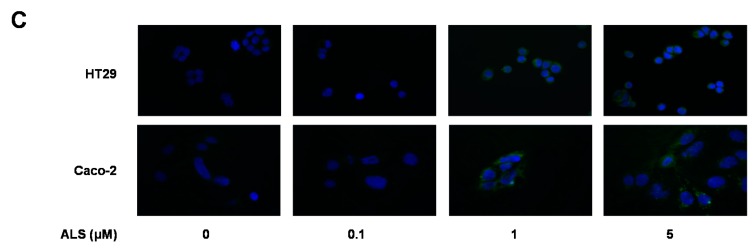
ALS induces autophagic cell death in HT29 and Caco-2 cells. (**A**) Cells were treated with ALS at concentrations of 0.1, 1, and 5 µM for 24 h and cell samples were subject to flow cytometry analysis. Flow cytometric dot plots showing autophagic HT29 and Caco-2 cells stained by Cyto-ID^®^; (**B**) HT29 and Caco-2 cells were treated with ALS at 1 µM for 4, 8, 12, 24, 48, and 72 h and then subject to flow cytometry analysis. Flow cytometric dot plots showing autophagic HT29 and Caco-2 cells stained by Cyto-ID^®^; (**C**) HT29 and Caco-2 Cells were treated with ALS at 0.1, 1, and 5 µM for 24 h. Cells were stained with green fluorescent Cyto-ID^®^ and subjected to confocal microscopy to detect autophagy. Confocal microscopic images showing autophagy in HT29 and Caco-2 cells. The box indicates the events that were counted. FL: fluoresence. Maganification: 40×.

As there was a clear ALS-induced apoptosis in HT29 and Caco-2 cells, we next investigated the effect of ALS on the autophagy of HT29 and Caco-2 cells by flow cytometric analysis and confocal microscopic examination. As shown in Figure 6A and Figure S6A, treatment of HT29 and Caco-2 cells with ALS for 24 h induced autophagy in a concentration-dependent manner. The percentage of intracellular autophagic signal at the basal level was 6.9% and 9.6% for HT29 and Caco-2 cells, respectively. Incubation of HT29 cells with 1 and 5 μM ALS for 24 h resulted in a 5.3- and 6.1-fold increase in the percentage of autophagic signal, respectively, compared to the control cells (*p* < 0.01; Figure 6A and Figure S6A). For Caco-2 cells, incubation with 1 and 5 μM ALS for 24 h resulted in a 1.7- and 2.0-fold increase in the percentage of autophagic signal, respectively, compared to the control cells (*p* < 0.01; Figure 6A and Figure S6A).

Furthermore, the effect of ALS on intracellular autophagy of HT29 and Caco-2 cells over time-course was evaluated. Both cells were exposed to ALS at 1 μM for 4, 8, 12, 24, 48, and 72 h. The percentage of autophagic signal was increased 3.2-, 5.6-, and 7.0-fold when HT29 cells were treated for 24, 48, and 72 h, respectively, compared to control cells (*p* < 0.001; Figure 6B and Figure S6B). For Caco-2 cells, the percentage of autophagic signal was increased 2.0-, 3.0-, and 2.5-fold when cells were treated for 24, 48, and 72 h, respectively, compared to control cells (*p* < 0.001; Figure 6B and Figure S6B). These results indicate that ALS induces a remarkable autophagy of HT29 and Caco-2 cells in concentration- and time-dependent manners.

Next, we performed the confocal microscopy to determine the autophagy-inducing effect of ALS in HT29 and Caco-2 cells. As shown in Figure 6C and Figure S6C, in comparison to the control cells, there was a 2.2- and 3.1-fold increase in the autophagy level of HT29 cells when treated with ALS at 1 and 5 μM for 24 h, respectively (*p* < 0.001). For Caco-2 cells, treatment with ALS at 1 and 5 μM for 24 h caused a 1.9- and 2.2-fold increase in the autophagy level when compared with the control cells, respectively (*p* < 0.001; Figure 6C and Figure S6C). There was no significant change in the autophagy level when both cell lines were treated with ALS at low concentration (0.1 µM). These results show that ALS treatment induces a significant concentration-dependent increase in autophagy of HT29 and Caco-2 cells.

### 2.8. ALS Regulates PI3K/Akt/mTOR Axis, AMPK, and p38 MAPK Signaling Pathways in HT29 and Caco-2 Cells

**Figure 7:**
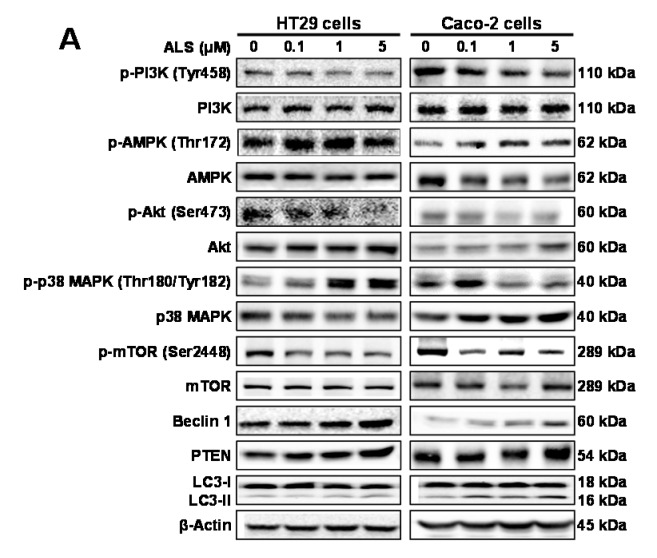

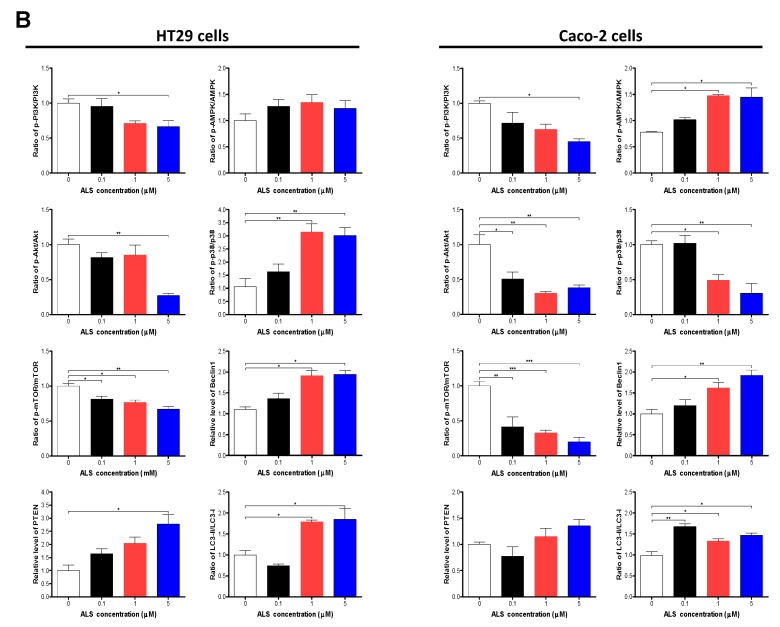
Effect of ALS on the expression or phospharylation levels of key autophagy-regulating molecules in HT29 and Caco-2 cells. Cells were treated with ALS at 0.1, 1, and 5 µM for 48 h. The phosphorylation level of PI3K, AMPK, Akt, p38 MAPK, and mTOR, and the total level of mTOR, beclin 1, PTEN, LC3-I, and LC3-II in HT29 and Caco-2 cells determined by Western blotting assay. (**A**) Representative blots of p-PI3K, PI3K, p-AMPK, AMPK, p-Akt, Akt, p-p38 MAPK, p38 MAPK, p-mTOR, mTOR, beclin 1, LC3-I, and LC3-II in HT29 and Caco-2 cells treated with ALS for 48 h; and (**B**) Bar graphs showing the ratio of p-PI3K/PI3K, p-AMPK/AMPK, p-Akt/Akt, p-p38 MAPK/p38 MAPK, p-mTOR/mTOR, LC3-II/LC3-I, and the expression levels of beclin 1 and PTEN in HT29 and Caco-2 cells treated with ALS for 48 h. β-Actin was used as the internal control. Data are expressed as the mean ± SD of three independent experiments. * *p* < 0.05, ** *p* < 0.01, and *** *p* < 0.001 by one-way ANOVA.

After determining the autophagy-inducing effect of ALS in HT29 and Caco-2 cells, we further elucidated the latent mechanism for the ALS-induced autophagy. ALS treatment results in remarkable changes in the expression and phosphorylation levels of key functional molecules involved in autophagy signaling pathway. First, we assessed the phosphorylation level of PI3K at Tyr199, AMPK at Thr172, and p38 MAPK at Thr180/Tyr182. These proteins execute critical roles in the regulation of cell proliferation, cell survival, cell migration and cell death as the upstream signaling molecules of the protein kinase B (Akt)/mammalian target of rapamycin (mTOR) pathway [24,25,26]. Exposure of HT29 cells to ALS for 48 h declined the phosphorylation level of PI3K (Tyr199) and increased the expression level of total PI3K (Figure 7A,B). Consequently, the p-PI3K/PI3K ratio was decreased 4.4%, 29.1%, and 33.7% (*p* < 0.001; Figure 7A,B) when treated with ALS at 0.1, 1, and 5 μM, respectively, compared to control cells. The p-AMPK/AMPK ratio was increased 1.3-, 1.3-, and 1.2-fold when HT29 cells were treated with ALS at 0.1, 1, and 5 μM, respectively, compared to control cells (Figure 7A,B); while the p-p38/p38 ratio was increased 1.6-, 3.1-, and 3.0-fold when HT29 cells were treated with ALS at 0.1, 1, and 5 μM, respectively, compared to control cells (*p* < 0.05 or 0.01; Figure 7A,B).

As a downstream effector of PI3K, Akt plays a key role in regulating diverse cellular functions including metabolism, cell proliferation, survival, growth, migration, invasion, and angiogenesis [27]. The mTOR kinase is a downstream target of Akt, which is phosphorylated and activated at Ser473 by mTORC2 and at Thr308 by 3-phosphoinositide-dependent protein kinase-1 [28]. mTOR is regulated by signaling pathway originating from starvation, growth factors, and cellular stressors, and plays a critical role in cell growth, autophagic cell death, and homeostasis [29]. We next evaluated the phosphorylation of Akt at Ser473 and mTOR at Ser2448 after ALS treatment of HT29 cells. There was a concentration-dependent decline in the phosphorylation level of Akt and mTOR with ALS treatment at 0.1, 1, and 5 µM (Figure 7A,B). However, the expression of total Akt and mTOR was not significantly altered after ALS incubation (Figure 7A,B). Thus, in comparison to the control cells, the ratio of p-Akt over Akt was markedly reduced 18.6%, 14.8%, and 72.8% and the ratio of p-mTOR over mTOR was declined 18.4%, 23.3%, and 33.1% when HT29 cells were treated with ALS at 0.1, 1, and 5 μM, respectively (*p* < 0.01; Figure 7A,B). PTEN is a dual-specificity phosphatase and tumor suppressor gene, acting as a negative regulator of Akt/mTOR and MAPK signaling, with a key function of cell death [30]. Therefore, we examined the expression level of PTEN when HT29 cells were treated with ALS for 48 h. Exposure of HT29 cells to ALS at 0.1, 1, and 5 µM resulted in a 1.6-, 2.0-, and 2.8-fold increase in the expression level of PTEN, respectively, compared to the control cells (*p* < 0.01; Figure 7A,B).

Beclin 1 and LC3 are two important markers of vesicle expansion and formation during the autophagy process. Thus, we examined the effect of ALS on expression of beclin 1, LC3-I, and LC3-II in HT29 cells. There was a significant increase in the expression of beclin 1 and ratio of LC3-II over LC3-I when cells were treated with ALS (Figure 7A,B). In comparison with the control cells, the expression level of beclin 1 was increased 1.6- and 1.9-fold with the treatment of ALS at 1 and 5 µM, respectively, and the level of LC3-II was increased after ALS treatment in HT29 cells. The ratio of LC3-II/LC3-I was increased 1.8- and 1.8-fold when cells were treated with ALS at 1 and 5 μM, respectively (*p* < 0.01; Figure 7A,B). These results indicate that inhibition of the PI3K/Akt/mTOR pathway and activation of AMPK and p38MAPK contribute to the autophagy-inducing effect of ALS on HT29 cells.

For Caco-2 cells, incubation with ALS for 24 h led to marked alteration in the phosphorylation level of PI3K, p38 MAPK, and AMPK, but did not affect the total expression level (Figure 7A,B). There was a 37.4% and 54.8% decline in the ratio of p-PI3K over PI3K, a 51.0% and 69.6% decrease in the ratio of p-p38 over p38, and a 1.9- and 1.9-fold increase in the ratio of p-AMPK over AMPK when treated with ALS at 1 and 5 µM, respectively (Figure 7A,B). We further assessed the change in the phosphorylation of Akt at Ser473 and mTOR at Ser2448. In comparison with the control cells, there was a concentration-dependent decrease in the phosphorylation level of Akt and mTOR, while there was no significant alteration in the total expression level of Akt and mTOR when treated with ALS at 0.1, 1, and 5 µM (Figure 7A,B). Consequently, compared to the control cells, there was a 49.6%, 69.9%, and 61.8% decrease in the ratio of p-Akt over Akt (*p* < 0.01; Figure 7A,B), and a 58.7%, 67.5%, and 79.9% reduction in the ratio of p-mTOR over mTOR in Caco-2 cells incubated with ALS at 0.1, 1, and 5 µM, respectively (*p* < 0.001; Figure 7A,B). We also observed the expression level of PTEN after ALS incubation. In comparison with the control cells, there was a 1.2- and 1.4-fold increase in the expression level of PTEN in Caco-2 cells exposed to ALS at 1 and 5 µM, respectively (Figure 7A,B). Finally, we evaluated the expression levels of beclin 1, LC3-I, and LC3-II in Caco-2 cells treated with ALS. ALS caused a prominent increase in the expression levels of beclin 1 and LC3-II, but did not caused alteration in the expression level of LC3-I (Figure 7A,B). In comparison to the control cells, there was a 1.6- and 1.9-fold increase in the expression level of beclin 1 when treated with ALS at 1 and 5 µM, respectively; and there was a 1.7-, 1.4-, and 1.5-fold increase in LC3-II/LC3-I ratio when treated with ALS at 0.1, 1, and 5 µM, respectively (*p* < 0.01; Figure 7A,B). These findings show that inhibition of PI3K/Akt/mTOR pathway, suppression of p38MAPK, and activation of AMPK contribute to the autophagy-inducing effect of ALS on Caco-2 cells.

### 2.9. There Is a Crosstalk between ALS-Induced Apoptosis and Autophagy in HT29 and Caco-2 Cells

**Figure 8:**
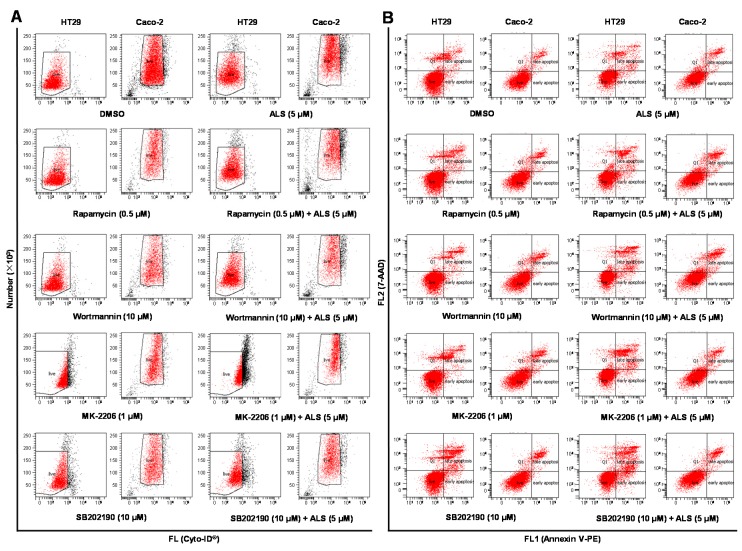
Effect of various inducers and inhibitors on the apoptosis and autophagy induced by ALS in HT29 and Caco-2 cells. The cells were pretreated with each of the compounds for 1 h before ALS was added and incubated for a further 24 h. Cells were double stained with Annexin V:PE (phycoerythrin) and 7-Aminoactinomycin D (7-AAD) to detect cellular apoptosis, after the cells were treated with ALS for 24 h. The autophagy was detected using the Cyto-ID^®^ green fluorescent dye to stain autophagy-associated vacuoles. (**A**) Flow cytometric dot plots showing the effects of a series of compounds on basal and ALS-induced apoptosis in HT29 and Caco-2 cells; (**B**) Flow cytometric dot plots showing the effects of the compounds on basal and ALS-induced autophagy in HT29 and Caco-2 cells. The box indicates the events that were counted. DMSO: dimethyl sulfoxide.

To further dissect the crosstalk between autophagy and apoptosis in HT29 and Caco-2 cells responding to ALS treatment, the flow cytometry was used to simultaneously examine cellular autophagy and apoptosis. First, we assessed the effect of induction or inhibition of autophagy on basal and ALS-induced autophagy in both cell lines. In HT29 cells, incubation with 0.5 µM rapamycin (a mTOR inhibitor and autophagy inducer ), 10 µM WM (a PI3K inhibitor and autophagy blocker), 1 µM MK2206 (a selective inhibitor of Akt and autophagy inducer), or 10 µM SB202190 (a selective p38 MAPK inhibitor and autophagy inducer) alone for 24 h led to a 5.4-, 4.3-, 2.0-, or 3.7-fold increase in basal autophagy compared to the control cells, respectively (*p* < 0.001; Figure 8A and Figure S7). Co-incubation with 0.5 µM rapamycin or 10 µM SB202190 increased ALS-induced autophagy 1.9- or 1.9-fold in HT29 cells, respectively (*p* < 0.001; Figure 8A and Figure S7), while co-incubation with 10 µM WM or 1 µM MK2206 only slightly enhanced ALS-induced autophagy. In Caco-2 cells, treatment with 0.5 µM rapamycin, 1 µM MK2206, or 10 µM SB202190 alone for 24 h induced a 2.7-, 6.5-, or 3.4-fold increase in basal autophagy compared to the control cells, respectively (*p* < 0.001; Figure 8A and Figure S7), while 10 µM WM diminished the basal autophagy. Pre-incubation with 0.5 µM rapamycin, 1 µM MK2206, or 10 µM SB202190 increased ALS-induced autophagy 1.5-, 3.1-, or 1.9-fold in Caco-2 cells (*p* < 0.001; Figure 8A and Figure S7). Of note, pre-treatment with 10 µM WM significantly decreased ALS-induced autophagy (Figure 8A and Figure S7).

Next, we evaluated the effect of induction or inhibition of autophagy on basal and ALS-induced apoptosis in HT29 and Caco-2 cells. Incubation of HT29 cells with 10 µM SB202190 alone for 24 h remarkably induced apoptosis (9.6% *vs.* 19.4%, *p* < 0.05 Figure 8B and Figure S7), while incubation of the cells with 0.5 µM rapamycin, 10 µM WM, or 1 µM MK2206 for 24 h had no significant effect on the basal apoptosis of HT29 cells (Figure 8B and Figure S7). In addition, co-incubation of HT29 cells with 10 µM SB202190 increased ALS-induced apoptosis (14.4% *vs.* 23.7%, *p* < 0.001; Figure 8B and Figure S7), whereas co-incubation with 0.5 µM rapamycin, 10 µM WM, or 1 µM MK2206 only slightly altered ALS-induced apoptosis. In Caco-2 cells, exposure to 0.5 µM rapamycin, 10 µM WM, 10 µM SB202190 or 1 µM MK2206 alone only slightly increased the basal apoptosis (Figure 8B and Figure S7). Pre-incubation with 10 µM WM enhanced ALS-induced apoptosis (9.7% *vs.* 14.0%, *p* < 0.001; Figure 8B and Figure S7), whereas co-incubation with 0.5 µM rapamycin, 1 µM MK2206, or 10 µM SB202190 only slightly altered ALS-induced apoptosis (Figure 8B and Figure S7). Taken together, these data shows that regulation of p38 MAPK by SB202190 may alter ALS-induced autophagy and apoptosis in HT29 cells and modulation of PI3K by WM may alter ALS-induced autophagy and apoptosis in Caco-2 cells with differential effects depending on the cell type. The autophagy inducer SB202190 enhances the ALS-induced apoptosis in both HT29 and Caco-2 cells, whereas the autophagy blocker WM increases ALS-induced autophagy in HT29 cells but decreases it in Caco-2 cells.

### 2.10. ALS Suppresses EMT in HT29 and Caco-2 Cells

**Figure 9:**
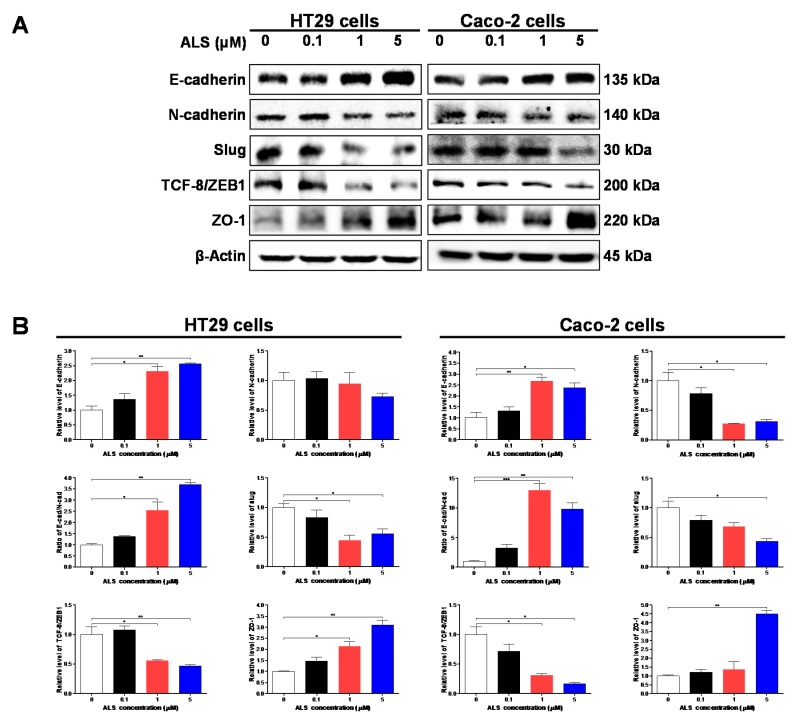
Effect of ALS on expression levels of epithelial to mesenchymal transition (EMT) associated markers in HT29 and Caco-2 cells. Cells were treated with ALS at concentrations of 0.1, 1, and 5 µM for 48 h and then protein samples were subject to Western blotting assay. (**A**) Representative blots showing the expression level of E-cadherin, N-cadherin, slug, TCF-8/ZEB1, and ZO-1 in HT29 and Caco-2 cells determined by Western blotting assay; (**B**) Bar graphs showing the expression level of E-cadherin, N-cadherin, slug, TCF-8/ZEB1, and ZO-1, and the ratio of E-cadherin over N-cadherin in HT29 and Caco-2 cells. β-Actin was used as the internal control. Data represent as the mean ± SD of three independent experiments. * *p* < 0.05, ** *p* < 0.01, and *** *p* < 0.001 by one-way ANOVA.

Epithelial-to-mesenchymal transition (EMT) is a critical event in which epithelial cells trans-differentiate into fibroblastic migratory cells through losing intracellular adhesion and cellular polarity and obtaining mesenchymal properties and increased motility. It has been suggested that EMT contributes to the cancer cells spreading and migrating to distant organs to form metastases [31,32]. E-cadherin is a hallmarker of the epithelial phenotype while N-cadherin is considered a key mesenchymal phenotype. Loss of E-cadherin and increasing N-cadherin expression facilitates the occurring of EMT and metastasis [33]. We speculated that ALS can inhibit EMT phenotype in HT29 and Caco-2 cells. So we examined the expression of EMT-associated regulators, including E-cadherin, N-cadherin, slug, TCF-8, and ZO-1. First, ALS incubation resulted in remarkable effect on the expression level of E-cadherin and N-cadherin (Figure 9A,B). Treatment of HT29 cells with ALS at 1 and 5 µM led to a 2.3- and 2.6-fold increase in the expression level of E-cadherin, respectively, compared to the control cells (*p* < 0.01; Figure 9A,B), but treatment of HT29 cells with ALS at 0.1, 1, and 5 µM resulted in a 3.4%, 5.3%, and 27.4% decrease in the expression level of N-cadherin, respectively (Figure 9A,B). Although there was no significant difference in the expression of N-cadherin, an increased ratio of E-cadherin over N-cadherin was observed. The ratio of E-cadherin/N-cadherin was increased 1.4-, 2.5-, and 3.7-fold when HT29 cells were exposed to ALS at 0.1, 1, and 5 µM for 48 h, respectively (*p* < 0.01; Figure 9A,B). In Caco-2 cells, in comparison to the control cells, the expression level of E-cadherin was increased by 2.7- and 2.7-fold when cells were treated with ALS at 1 and 5 μM, respectively, (*p* < 0.01; Figure 9A,B). Meanwhile, the expression level of N-cadherin was decreased by 46.4%, 72.8%, and 69.1% when cells were incubated with ALS at 0.1, 1, and 5 μM, respectively, (*p* < 0.01; Figure 9A,B). The ratio of E-cadherin/N-cadherin was increased 3.2-, 13.0-, and 9.8-fold when Caco-2 cells were incubated with ALS at 0.1, 1, and 5 μM for 48 h, respectively, (*p* < 0.01; Figure 9A,B).

Slug is a zinc finger transcriptional factor, together with TCF8/ZEB1 acting as suppressors of E-cadherin in EMT [34]. We further examined the effect of ALS incubation of HT29 and Caco-2 cells on the expression of slug and TCF-8/ZEB1. As shown in Figure 9A,B, there was a significant reduction in the expression levels of slug and TCF-8/ZEB1. In comparison with control cells, exposure of HT29 cells to ALS at 1 and 5 µM led to a 55.3% and 44.1% decline in the expression level of slug, respectively (*p* < 0.05; Figure 9A,B). Exposure of HT29 cells to ALS at 1 and 5 µM resulted in a 44.5% and 53.4% reduction in the expression level of TCF-8/ZEB1, respectively (*p* < 0.001; Figure 9A,B). Similarly, in Caco-2 cells, treatment of cells to ALS led to a concentration-dependent reduction in the expression level of slug and TCF-8/ZEB1 (*p* < 0.05 or 0.01; Figure 9A,B).

In the end, we assessed the effect of ALS on expression of ZO-1 in HT29 and Caco-2 cells. ZO-1 is a member of Zonula occludens proteins which are involved in the regulation of cytoskeletal organization, signal transduction, transcriptional modulation and cell polarity maintenance. ZO-1 are required for tight junction formation and function. It has been suggested that the cytoplasmic/nuclear relocalization of β-catenin and ZO-1 from the adhesions and tight junctions are common processes of the epithelial-mesenchymal transition associated with tumor invasion [35]. As shown in Figure 9, ALS significantly increased the expression level of ZO-1 in both cell lines. Compared to the control cells, there was a 2.1- and 3.1-fold increase in the expression level of ZO-1 when HT29 cells were treated with ALS at 1 and 5 µM, respectively (*p* < 0.01; Figure 9A,B). For Caco-2 cells, there was a 1.2-, 1.4-, and 4.5-fold increase in the expression level of ZO-1 when treated with ALS at 0.1, 1, and 5 µM respectively, compared to the control cells (*p* < 0.01; Figure 9A,B). In aggregate, all these results demonstrate that ALS has a strongly inhibitory effect on EMT by upregulating the expression level of E-cadherin and ZO-1, and down-regulating the expression level of N-cadherin, slug, and TCF-8 in HT29 and Caco-2 cells.

## 3. Discussion

Due to the high mortality and morbidity resulted from CRC worldwide, CRC places a substantial heavy burden on individual and society [36]. It requires novel therapeutic agent with more therapeutic efficacy and less severe side effects. In particular, patients with advanced stages of CRC often suffer from the recurrence, metastasis, and complications, and frequently display poor therapeutic outcome, despite the application of various therapeutic strategies, including potentially curable surgery, chemotherapy, selective radiotherapy and other forms of treatment. Recently, compelling evidence indicates that AURKA can act as a novel and promising antineoplastic target; its selective inhibitor, ALS, is a small molecule which has demonstrated the potent anticancer effect in the treatment of various types of cancer in preclinical and clinical studies [15,16,17,19,20,21,22,37]. In the present study, a potent anti-proliferative effect of ALS was observed in HT29 and Caco-2 cells. We have found that ALS exerts a remarkable inducing effect on cell cycle arrest in G_2_/M phase and markedly promotes apoptosis and autophagy in concentration- and time-dependent manners in both cell lines with the involvement of multiple signaling pathways, including mitochondrial pathway, death receptor signaling pathway, PI3K/Akt/mTOR, p38 MAPK, and AMPK signaling pathways. Moreover, the inhibitory effect of ALS on the EMT may also contribute to its antitumor potential.

The cell cycle regulatory proteins, which have been found involved in proliferation of tumor cells, including the cyclins, the cyclin dependent kinases (CDKs), their downstream substrates, the CDK inhibitors (CKIs), and the tumor suppressor proteins (e.g., p53 and Rb). Activated CDK1 (CDC2) bound to cyclin B1 promotes entry into M-phase in cell cycle. So it is pivotal to maintain the activity of the cyclin B1-CDC2 (CDK1) complex in promoting G_2_/M transition process. There are many upstream regulators modifying the activity of the cyclin B1-CDC2 (CDK1) complex. p53 is a critical tumor suppressor protein and a positive regulator of cell cycle arrest. Normally, the G_2_/M DNA damage or chromosomal instability promote the phosphorylation of p53 and accelerates its dissociation from MDM2 and MDM4. Consequently, the transcriptional ability of p53 is activated. The p53 downstream-regulated genes including 14-3-3, p21, GADD45, and WIP1 ultimately serve to inactivate the Cyclin B1-CDC2 complex and suppress the entry into G_2_/M transition. AURKA is localized at the mitotic spindle and centrosome, where it interacts with many functional proteins/substrates, including PP1, p53, Cdh-1, TPX-2, RasGAP, and Ajuba. It was reported that in the absence of hyperactivation of AURAK, the crosstalk of AURKA with p53 caused tumorigenesis transformation of cells by counteracting the p53 mediated tumor suppression [38]. In our work, we detected a potent effect of ALS on G_2_/M phase cell cycle arrest in HT29 and Caco-2 cells, and we found that the active form of p-AURKA at Thr288 was significantly suppressed by ALS. There was an increase in expression level of p53 and p21 and a decrease in expression level of cyclinB1 and CDC2 which were observed as well. Collectively, these results indicate that ALS-induced G_2_/M cell cycle arrest may be attributed to its inhibitory effect on the interaction of AURKA and p53, however, this explanation needs to be further verified for CRC treatment.

On the other hand, due to the lack of p53 expression in Caco-2 cells, we, therefore, examined the upstream molecules regulating the cyclin B1-CDC2 (CDK1) complex. PLK1 could be activated by the concerted function of AURKA and the cofactor Bora [39]. CDC25 that can be activated by PLK1 and inactivated by Chk kinases through phosphorylation, is a positive regulator of CDC2 by removal of phosphates at Thr14 and Tyr15 [40,41,42]. In addition, PLK1 can phosphorylate cyclin B1 at Ser133 resulting in a rapid nuclear import of cyclin B1, which promotes the mitosis entry [43]. In the present study, we found a significant increase in the level of p-CDC25C (Ser216), total cyclinB1, and p-CDC2 (Tyr 15), but a decline in the level of PLK1, p-cyclinB1 (Ser133), suggesting that the PLK1-CDC25C-cyclinB1 and CDC2 complex axis is involved in ALS-induced G_2_/M cell cycle arrest in Caco-2 cells. Also, we also found that ALS induced cell cycle arrest in G_2_/M phase in gastric cancer cells [19], pancreatic cancer cells [21], osteosarcoma cells [22], breast cancer cells [44], and ovarian cancer cells [20].

Apoptosis is a distinct genetic and biochemical pathway of cell death necessary for cell growth, development and maintenance of homeostasis in metazoans. There are two specific pathways activating cell apoptosis, including intrinsic and extrinsic death pathway [45,46]. In this study, cytochrome c was released from mitochondria after ALS treatment, which might be caused by the increased expression level of Bax and decreased expression level of Bcl-2 and Bcl-xl. Subsequently, caspase 9 was activated with the elevated level of cleaved caspase 9, which in turn cleaved caspase 3 and PARP and ultimately induced apoptosis. The expression level of PUMA, which acted as one of pro-apoptotic members of Bcl-2 family, was increased in a concentration-dependent manner. These data indicate that ALS induces mitochondria-dependent apoptosis of HT29 cells. For Caco-2 cells, a decrease in the expression level of Bax and increased expression level of Bcl-2 were observed after ALS treatment, which suggests that mitochondrial pathway might be not involved in the ALS-induced apoptosis. Then, we found a significant increase in the expression level of RIP and the p-FADD/FADD ratio, which might initiate the death receptor signaling pathway. Consequently, caspase cascades was activated, including activated caspase-8 and cleaved caspase-3. Therefore, ALS induces apoptosis of Caco-2 cells via death receptor signaling pathway. Additionally, in comparison to previous studies on the apoptosis inducing effect of ALS, it showed that ALS induced mitochondria-dependent apoptosis cell death in gastric cancer cells [19], pancreatic cancer cells [21], osteosarcoma cells [22], breast cancer cells [44], and ovarian cancer cells [20], showing the most potent cancer cell killing effect in pancreatic cancer cell lines PANC-1 and BxPC-3 [21].

Autophagy is a cellular process of catabolic degradation in which damaged, dysfunctional, or superfluous organelles and proteins are sequestered, engulfed, and recycled to maintain cellular metabolism, viability and homeostasis [25]. Autophagy is generally induced by the deprivation of nutrient or stress. The mTOR kinase, which is activated by signaling pathway originating from growth factors and nutrient availability but inhibited in response to starvation, plays a critical role in regulating autophagy progression. Suppression of mTOR is required for induction of autophagy by limiting the inhibitory effect on the ULK1 kinase complex [47]. There are diverse signaling pathways implicated in the regulation of mTOR signaling, including positive regulation of mTOR (PI3K/Akt and p38 MAPK signaling) suppressing autophagy and negative regulation of mTOR (AMPK and p53 signaling) promoting autophagy [48,49,50]. The role of autophagy in tumorigenesis is controversial. Under certain circumstances, autophagy suppresses tumorigenesis by increasing programmed cell death [51]. However, in other cases, autophagy provides cancer cells with a rescue mechanism to sustain cell viability [52]. The present study showed a remarkable autophagy-inducing effect of ALS on HT29 and Caco-2 cells. The underlying mechanism of this autophagy-inducing effect may be ascribed to the inhibition of PI3K/Akt/mTOR pathway and activation of AMPK in both cell lines. Interestingly, there is a differential alteration in the modification of p38 MAPK signaling pathway. This phenomenon may be associated with the existence of four isoforms of p38 MAPK (p38α, p38β, p38γ, and p38δ), which differ in their tissue distribution profile, upstream regulators and downstream targets in a cell type- and stimulus-dependent manner in the mammals [49]. In addition, HT29 cells do not have mutations in the *p53* gene, while Caco-2 cells have a mutated *p53* gene without *p53* expression. These different characteristics may also contribute to the differential alteration in the regulation of p38 MAPK signaling pathway. On the other hand, the previous studies showed that ALS inhibited PI3K/Akt/mTOR, p38 MAPK, or Erk1/2 signaling pathways while activating AMPK signaling pathway, contributing to the pro-autophagic effects of ALS in gastric cancer cells [19], pancreatic cancer cells [21], osteosarcoma cells [22], breast cancer cells [44], and ovarian cancer cells [19,20,21].

Furthermore, autophagy and apoptosis are highly conserved and tightly regulated processes that play essential roles in development, tissue homeostasis and disease [53]. Generally, the two biological processes involved in similar regulatory pathways and even share initiator and effector molecules, which indicates a perplexing crosstalk. Accumulating evidence suggests that autophagy and apoptosis can cooperate in a balanced interplay to allow cells to decide which route to take, thus influencing differentially the fate of the cell. Our study showed a crosstalk between apoptosis and autophagy by using rapamycin (a mTOR inhibitor and autophagy inducer), WM (a PI3K inhibitor and autophagy blocker), MK2206 (a selective inhibitor of Akt and autophagy inducer), and SB202190 (a selective p38 MAPK inhibitor and autophagy inducer). Similar regulatory effect of the autophagy inducer SB202190 on the ALS-induced apoptosis were observed, whereas differential influence of other chemical modulators on apoptosis and autophagy were present in both HT29 and Caco-2 cells. It can be speculated that several important molecules or pathways coordinated and mediated the complex interplay between autophagy and apoptosis, including TOR kinase pathway, p53, beclin 1, and Akt. Herein, ALS can induce apoptosis and autophagy in a coordinated manner in HT29 and Caco-2 cells.

Additionally, EMT is a cellular process in epithelial cells changing their adhesive repertoire, polarized arrange and cytoskeletal organization, and acquiring mesenchymal characteristics and migratory and invasive properties [54]. When it comes to cancer cells, EMT represents the conversion of fully differentiated epithelial cells into poorly differentiated and invasive mesenchymal cells [55]. EMT involves numerous distinct genetic and epigenetic alterations, including the decline in expression of epithelial markers, such as E-cadherin, ZO-1, claudins, occludins, β-catenin, and cytokeratins, and the elevation in expression of mesenchymal markers, such as N-cadherin, vimentin, TCF-8/ZEB-1, and slug [56]. The present study showed an increase in the expression level of E-cadherin and a decrease in the expression level of N-cadherin in HT29 and Caco-2 cells, with an elevated ratio of E-cadherin over N-cadherin, which demonstrate that the “cadherin switch” was closed and EMT was suppressed. We also found a significant alteration in the expression of ZO-1, TCF-8/ZEB-1, and slug. The down-regulation of epithelial cell-cell adhesion molecule E-cadherin and the up-regulation of mesenchymal cell-cell adhesion molecule N-cadherin expression, which is called the “cadherin switch”, is a major hallmark of EMT in cancer cells [57,58,59]. Taken together, the findings suggest that inhibitory effect on EMT contributing to the beneficial effects of ALS in HT29 and Caco-2 cells.

**Figure 10:**
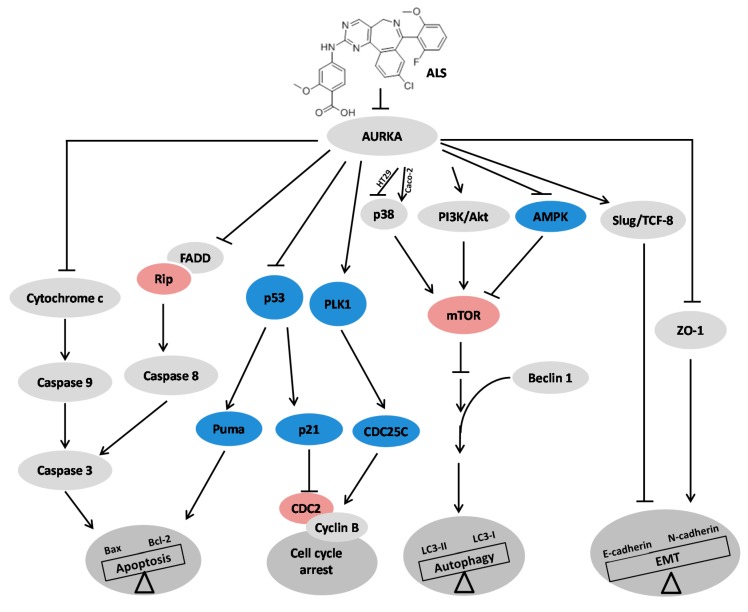
Schematic mechanism underlies the cancer cell killing effect of ALS in HT29 and Caco-2 cells.

In conclusion, in the present study, we have explored the anticancer effect of ALS and the potential molecular mechanisms for its cancer cell killing effect *in vitro*. The mechanism of actions of ALS includes the inhibition of cell proliferation, cell cycle arrest, activation of the mitochondria-dependent and death receptor-dependent apoptosis, and induction of autophagy in human HT29 and Caco-2 cells. Suppression of PI3K/Akt/mTOR and activation of AMPK signaling pathways are involved in the autophagy-inducing effect of ALS in HT29 and Caco-2 cells. In addition, the inhibitory effect on EMT contributes to the anticancer activity of ALS (Figure 10). In aggregate, ALS may represent a new targeted therapeutic agent that can kill CRC cells. However, more studies are warranted to fully delineate the underlying anticancer mechanism and the interaction of other potential targets of ALS in the treatment of colorectal cancer.

## 4. Materials and Methods

### 4.1. Chemicals and Reagents

ALS was bought from Selleckchem Inc. (Houston, TX, USA). DMSO, ^13^*C*_6_-l-lysine, l-lysine, ^13^*C*_6_,^15^*N*_4_-l-arginine, l-arginine, fetal bovine serum (FBS), dialyzed FBS, ammonium persulfate, propidium iodide (PI), ribonuclease (RNase A), protease and phosphatase inhibitor cocktails, MTT, bovine serum albumin, ethylenediaminetetraacetic acid (EDTA), and Dulbecco’s phosphate buffered saline (PBS) were sourced from Sigma-Aldrich (St Louis, MO, USA). FASP™ protein digestion kit was obtained from Protein Discovery Inc. (Knoxville, TN, USA). 8-well chamber slide was purchased from Lab-Tek^®^ Inc. (Hatfield, PA, USA). Dulbecco’s Modified Eagle’s Medium (DMEM) were sourced from Corning Cellgro Inc. (Herndon, VA, USA). 4’,6-Diamidino-2-phenylindole (DAPI), 5-(and 6)-chloromethyl-2’,7’-dichlorodihydrofluorescein diacetate (CM-H_2_DCFDA), wortmannin (WM; a selective inhibitor of PI3K and a blocker of autophagosome formation), MK2206 (a selective inhibitor of Akt), and SB202190 (4-(4-fluorophenyl)-2-(4-hydroxyphenyl)-5-(4-pyridyl)-^1^*H*-imidazole; a selective inhibitor of p38 MAPK used as an autophagy inducer) were obtained from InvivoGen Inc. (San Diego, CA, USA). Pierce™ bicinchoninic acid (BCA) protein assay kit, skim milk, radioimmunoprecipitation assay (RIPA) buffer, and Western blotting substrate were bought from Thermo Fisher Scientific Inc. (Waltham, MA, USA). The annexin V:PE (phycoerythrin) apoptosis detection kit was obtained from BD Biosciences Inc. (San Jose, CA, USA). Cyto-ID^®^ Autophagy detection kit was bought from Enzo Life Sciences Inc. (Farmingdale, NY, USA). Primary antibodies against human CDC2, phospho(p)-CDC2 (Tyr15), cyclin B1, p-cyclin B1 (Ser133), p-CDC25C (Ser216), p53, p21 Waf1/Cip1, Bax, Bcl-2, Bcl-xl, PUMA, cytochrome c, cleaved caspase 9, cleaved caspase 3, cleaved PARP, p-FADD (Ser194), FADD, RIP, p-PI3K (Tyr458), PI3K, p-p38 MAPK (Thr180/Tyr182), p38 MAPK, p-Akt (Ser473), Akt, p-mTOR (Ser2448), mTOR, beclin 1, and LC3-I/II were all purchased from Cell Signaling Technology Inc. (Beverly, MA, USA). The antibody against human β-actin was purchased from Santa Cruz Biotechnology Inc. (Dallas, TX, USA). The polyvinylidene difluoride (PVDF) membrane was bought from EMD Millipore Inc. (Billerica, MA, USA).

### 4.2. Cell Lines and Cell Culture

The HT29 and Caco-2 cells are two well-recognized CRC cell lines and were obtained from American Type Culture Collection (Manassas, VA, USA). These cells were maintained in DMEM supplemented with 10% heat-inactivated FBS and 1% penicillin/streptomycin in a 5% CO_2_/95% air humidified incubator at 37 °C. HT29 cells do not have mutations in the *p53* gene, while Caco-2 cells have a mutated *p53* gene without *p53* expression. ALS was dissolved in DMSO at a stock concentration of 100 mM and stored at −20 °C. The drug was freshly diluted with the final concentration of DMSO at 0.05% (volume per volume (*v*/*v*)). The control cells received 0.05% DMSO only.

For proteomic analysis, HT29 and Caco-2 cells were cultured in DMEM for SILAC (Sigma-Aldrich) with (heavy) or without (light) stable isotope labeled amino acids (^13^*C*_6_-l-lysine and ^13^*C*_6_,^15^*N*_4_-l-arginine) and 10% dialyzed FBS. Cells were cultured in SILAC medium for six cell doubling times to achieve a high level (>98%) of labeled amino acid incorporation. Then, the cells were grown in “light” media were treated with 0.5% DMSO for 48 h to function as the negative control; cells grown in “heavy” media were treated with predetermined ALS for 48 h. All the experiments were performed three times independently. After ALS treatment, cell samples were harvested, lysated with hot lysis buffer (100 mM Tris base, 4% sodium dodecyl sulfate (SDS) and 100 mM dithiothreitol), and denatured for 5 min at 95 °C. Then, the samples were stored at −80 °C till further analysis.

### 4.3. Proteomic Response to ALS Treatment Analyzed by Stable Isotope Labeling by Amino Acids in Cell Culture (SILAC)-Based Approach

#### 4.3.1. Digestion and Desalting SILAC Protein Samples

Prior to the quantitative proteomic analysis, the protein samples were subject to digestion and desalting which were performed using SILAC-based approach. Briefly, the thermal denatured cell lysate was sonicated, the supernatant was collected, and the protein concentration was determined. Subsequently, equal amount of heavy and light protein samples were combined to reach a total volume of 30–60 μL containing 300–600 μg protein. The combined samples were digested using FASP™ protein digestion kit according to the manufacturer’s instruction. After the digestion, the resultant sample was acidified to pH of 3 and desalted using a C_18_ solid-phase extraction column to desalt. The desalted samples were concentrated and resuspended in 0.1% formic acid prior to liquid chromatography-tandem mass spectrometry (LC-MS/MS) analysis.

#### 4.3.2. LC-MS/MS and Statistical Analysis

The concentrated samples (5 μL) were subject to the hybrid linear ion trap-Orbitrap (LTQ Orbitrap XL, Thermo Scientific Inc., Hudson, NH, USA). Peptide SILAC ratio was calculated using MaxQuant version 1.2.0.13 (Max Planck Institute of Biochemistry, Munich, Germany). The SILAC ratio was determined by averaging all peptide SILAC ratios from peptides identified of the same protein.

#### 4.3.3. Pathway and Network Analysis

The protein IDs were identified using Scaffold 4.3.2 from Proteome Software Inc. (Portland, OR, USA) and the pathway and network were analyzed using Ingenuity Pathway Analysis (IPA, www.ingenuity.com) from QIAGEN (Redwood City, CA, USA) as previously described [60].

### 4.4. Cell Viability Assay

The MTT assay was used to determine the effect of ALS on the viability of HT29 and Caco-2 cells. HT29 and Caco-2 cells were seeded into 96-well plates at a density of 6000 (Caco-2) or 8000 (HT29) cells/well. After cells were incubated for 24 h at a volume of 100 µL complete culture medium, the HT29 and Caco-2 cells were treated with ALS at concentrations ranging from 0.1 to 100 µM for 24 or 48 h. Following the ALS treatment, 10 µL of MTT stock solution (5 mg/mL) was added to each well and cultured for another 4 h. Then the solution was carefully aspirated and 100 µL of DMSO was added into each well. The plate was shaken for 10 min for crystal dissolution. Then the cell viability was examined by reduction of MTT. The absorbance was measured and the *IC*_50_ values were determined as previously described [61].

### 4.5. Cell Cycle Distribution Analysis

The effect of ALS on cell cycle distribution was tested using PI as a DNA stain to determine DNA content by flow cytometry as previously described [21]. In brief, HT29 and Caco-2 cells were seeded into 60 mm Petri dishes for attaching overnight. When the cells reached ~75% confluence and were then treated with ALS at concentrations of 0.1, 1, and 5 μM for 24 h. In separate experiments, HT29 and Caco-2 cells were treated with 1.0 μM ALS for 4, 8, 12, 24, 48, or 72 h. The collected cells were fixed and stained, then subject to flow cytometry. A total of 10,000 events were analyzed.

### 4.6. Quantification of Cellular Apoptosis

Following the examination of the effect of ALS on cell growth, the effect of ALS on cell apoptosis was determined and the percentage of apoptotic cells was quantified using the annexin V:PE apoptosis detection kit after the cells were treated with ALS at different concentrations (0.1, 1, and 5 μM) for 24 h. In separate experiments, cells were treated with 1 μM ALS for 4, 8, 12, 24, 48, or 72 h as previously described [61].

### 4.7. Quantification of Cellular Autophagy

To determine the effect of ALS on autophagy in HT29 and Caco-2 cells, cellular autophagy was examined using flow cytometry. Briefly, HT29 and Caco-2 cells were seeded into 60 mm Petri dishes for incubation overnight. The cells were treated with ALS at 0.1, 1, and 5 μM for 24 h when they reached ~75% confluence. Also, in separate experiments, HT29 and Caco-2 cells were treated with 1.0 μM ALS for 4, 8, 12, 24, 48, or 72 h as previously described [61].

### 4.8. Simultaneous Determination of Apoptosis and Autophagy Using Flow Cytometry

We further examined the two modes of programmed cell death simultaneously in order to investigate the potential crosstalk between ALS-induced apoptosis and autophagy. HT29 and Caco-2 cells were seeded into 60 mm Petri dishes. After incubation overnight, cells were pretreated with 10 μM WM (a PI3K inhibitor and autophagy blocker), 1 μM rapamycin (an mTOR inhibitor and autophagy inducer), 10 μM SB202190 (a selective inhibitor of p38 MAPK) or 1 μM MK2206 (a selective inhibitor of Akt) for 1 h, then co-treated with 5 μM ALS for further 24 h. At the end of treatment, the cells were trypsinized and centrifuged at 250× *g* for 5 min. The cells were divided into two samples of equal volume for detection of apoptosis and autophagy respectively. The subsequent steps follow the procedure mentioned above.

### 4.9. Confocal Fluorescence Microscopy

To further examine the cellular autophagy level, confocal microscopic examination was performed using a Cyto-ID^®^ autophagy detection kit as previously described [61]. Briefly, HT29 and Caco-2 cells were seeded into an 8-well chamber slide at 30% confluence. The cells were treated with ALS at 0.1, 1, and 5 µM for 24 h. Next, cells were incubated with 100 µL of dual detection reagents and examined using a TCS SP2 laser scanning confocal microscope (Leica, Wetzlar, Germany) at wavelengths of 405/488 nm.

### 4.10. Western Blotting Analysis

Western blotting assays were used to examine the expression level of key regulators involved in cell cycle, apoptosis, autophagy, and EMT processes as previously described [61]. Briefly, HT29 and Caco-2 cells were collected and lysed after 48-h treatment with ALS at 0.1, 1, and 5 μM. The supernatant was collected and the protein concentrations were measured using a Pierce™ BCA protein assay kit. Thirty microgram proteins were subjected to Western blotting assay. Visualization was performed using an enhanced chemiluminescence kit (Thermal Scientific, Waltham, MA, USA) and the blots were analyzed using Image Lab 3.0 (BioRad, Hercules, CA, USA). Protein expression level was normalized to the matching densitometric value of the internal control β-actin.

### 4.11. Statistical Analysis

Data are expressed as the mean ± standard deviation (SD). Multiple comparisons were assessed by one-way analysis of variance (ANOVA) followed by Tukey’s multiple comparison procedure. *p* < 0.05 was considered statistically significant. All the assays were performed in triplicate.

## Supplementary Materials

Supplementary materials can be found at http://www.mdpi.com/1422-0067/17/1/41/s1.
