# Supplementary material for: Alisertib Induces Cell Cycle Arrest, Apoptosis, Autophagy and Suppresses EMT in HT29 and Caco-2 Cells
Source: Int J Mol Sci. 2015 Dec 29;17(1):41. doi: 10.3390/ijms17010041 (PMC4730286; doi:10.3390/ijms17010041)
Supplement: Supplementary file 1 [file ijms-17-00041-s001.pdf]

Supplementary Materials: Alisertib Induces Cell Cycle Arrest, Apoptosis, Autophagy and Suppresses EMT in HT29 and Caco-2 Cells

Bao-Jun Ren, Zhi-Wei Zhou, Da-Jian Zhu, Yong-Le Ju, Jin-Hao Wu, Man-Zhao Ouyang, Xiao-Wu Chen and Shu-Feng Zhou

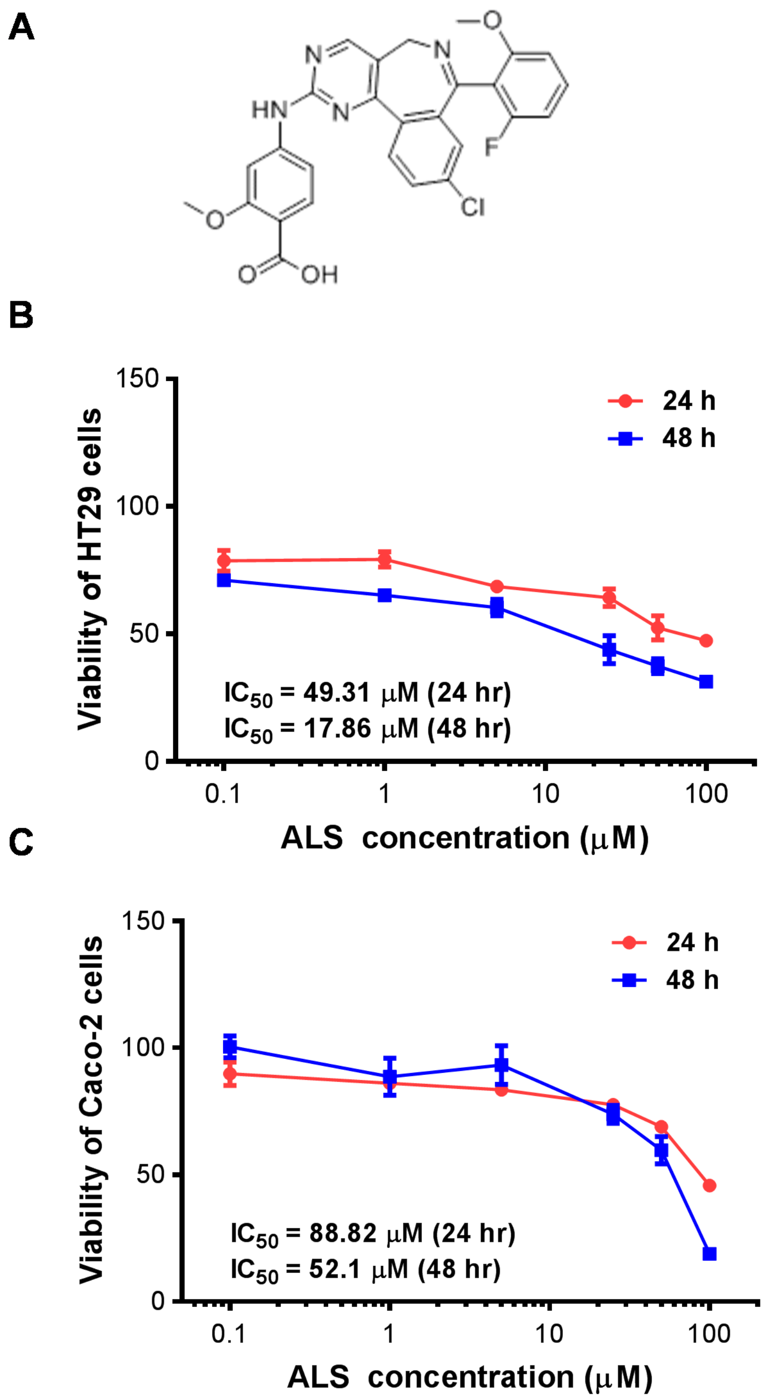

Figure Sl. The chemical structure of alisertib (ALS) and the viability of HT29 and Caco-2 cells after treatment of ALS. HT29 and Caco-2 cells were treated with ALS at concentrations ranging from 0.1 to 100 μM for 24 and 48 h. (**A**) The chemical structure of ALS and cell viability of HT29 (**B**) and Caco-2 (**C**) cells examined by 3-(4,5-dimethylthiazol-2-yl)-2,5-diphenyltetrazolium bromide (MTT) assay.

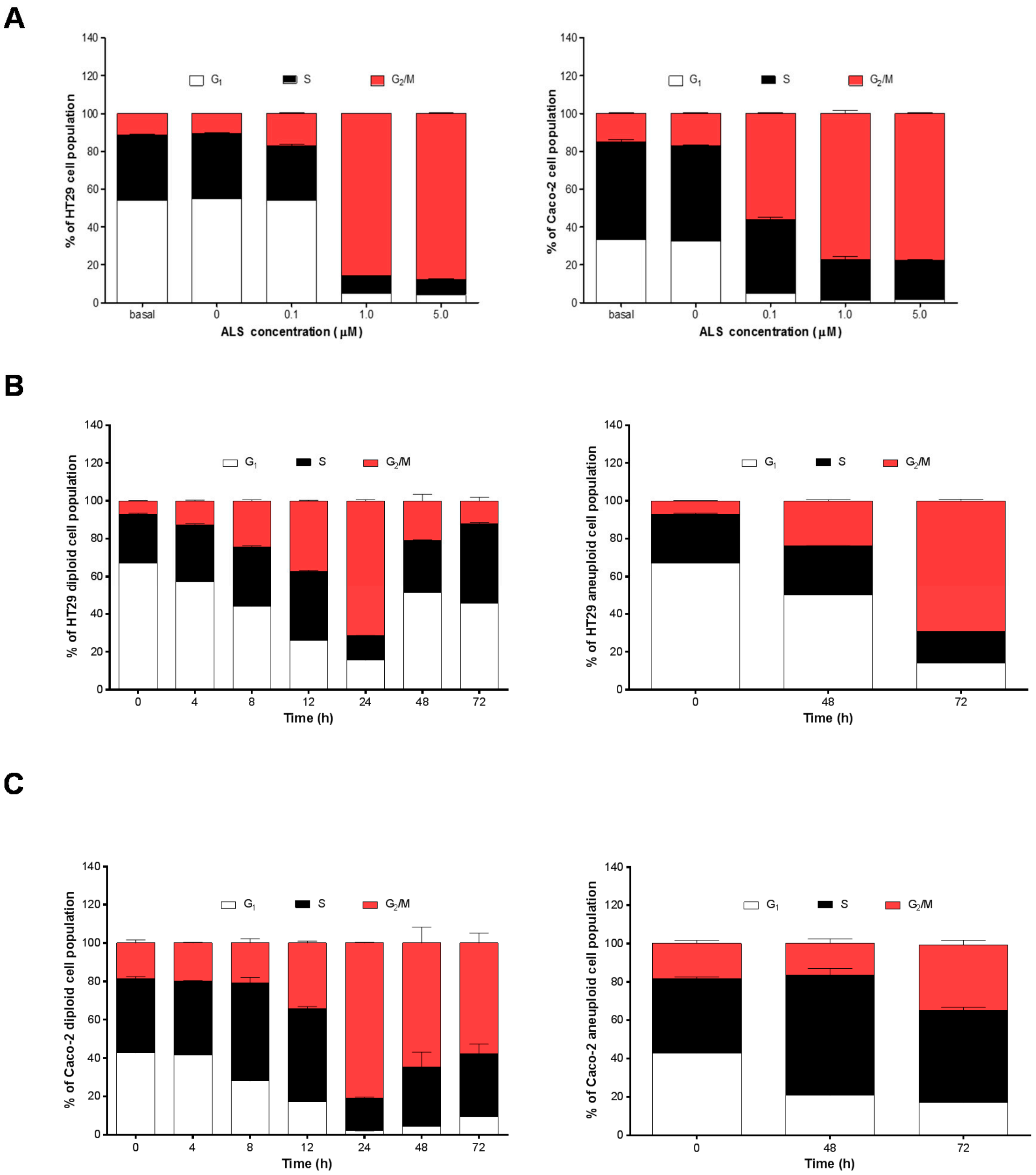

Figure S2. ALS induces cell cycle arrest in G_2/M phase in HT29 and Caco-2 cells. (**A**) HT29 and Caco-2 cells were treated with ALS at 0.1, 1, and 5 μM for 24 h and then subjected to flow cytometric analysis. The bar graphs showing the percentage of HT29 and Caco-2 cells in Gl, S, and G2/M phases; (**B**) Time course of ALS-induced cell cycle change over 72 h in HT29 cells. Bar graphs showing the cell cycle distribution when the cells were treated with ALS at 1 μM for 4, 8, 12, 24, 48, and 72 h; (**C**) Time course of ALS-induced cell cycle change over 72 h in Caco-2 cells. Bar graphs showing the cell cycle distribution when the cells were treated with ALS at 1 μM for 4, 8, 12, 24, 48, and 72 h. Cells were stained with PI and subjected to flow cytometric analysis that collected 15,000 events. Data represent the mean + SD of three independent experiments.

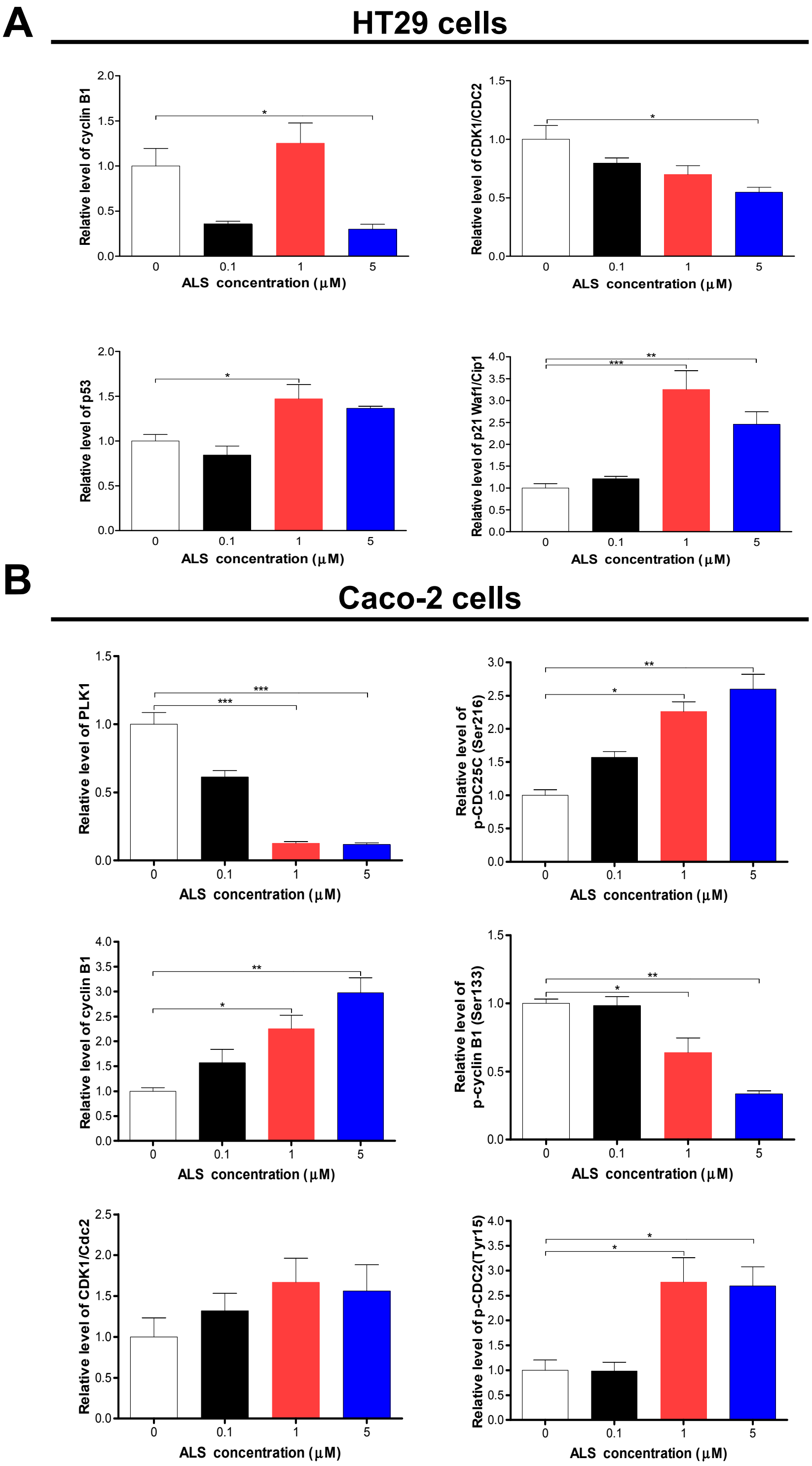

Figure S3. Effect of ALS on expression level of key regulators of cell cycle in HT29 and Caco-2 cells. (**A**) Bar graphs show the relative expression levels of CDK1/CDC2, cyclin Bl, p21 Waf1/Cip1, and p53 in HT29 cells; (**B**) Bar graphs showing the relative expression level of PLKI, p.CDC25C (ser216), cyclin 81, p-cyclin Bl (ser133), CDK1/CDC2, and p-CDC2 (Tyr15) in Caco-2 cells. Data are shown as the mean ± SD of three independent experiments. * $p < 0.05$, ** $p < 0.01$, and *** $p < 0.001$ by one-way analysis of variance (ANOVA).

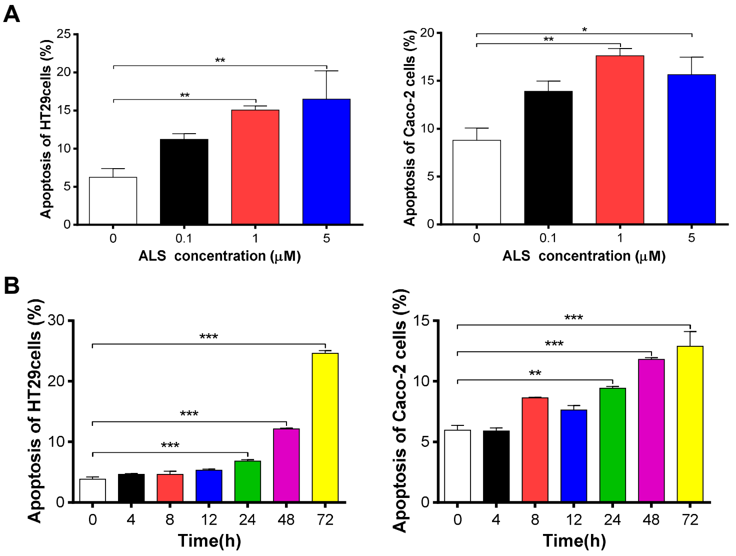

Figure S4. ALS induces apoptotic death in HT29 and Caco-2 cells. (**A**) H T 29 and Caco-2 cells were exposed to ALS at 0.1, 1, and 5 µM for 24 h and then subjected to flow cytometric analysis. Bar graphs showing the percentage of apoptotic cells in HT29 and Caco-2 cells; (**B**) H T 29 and Caco-2 cells were treated with ALS at 1 µM for 4, 8, 12, 24, 48, and 72 h and then subjected to flow cytometric analysis. Bar graphs showing the percentage of cells in the live, early apoptosis and late apoptosis stages in HT29 and Caco-2 cells. Data represent the mean + SD of three independent experiments. * $p < 0.05$, ** $p < 0.01$, and *** $p < 0.001$ by one-way ANOVA.

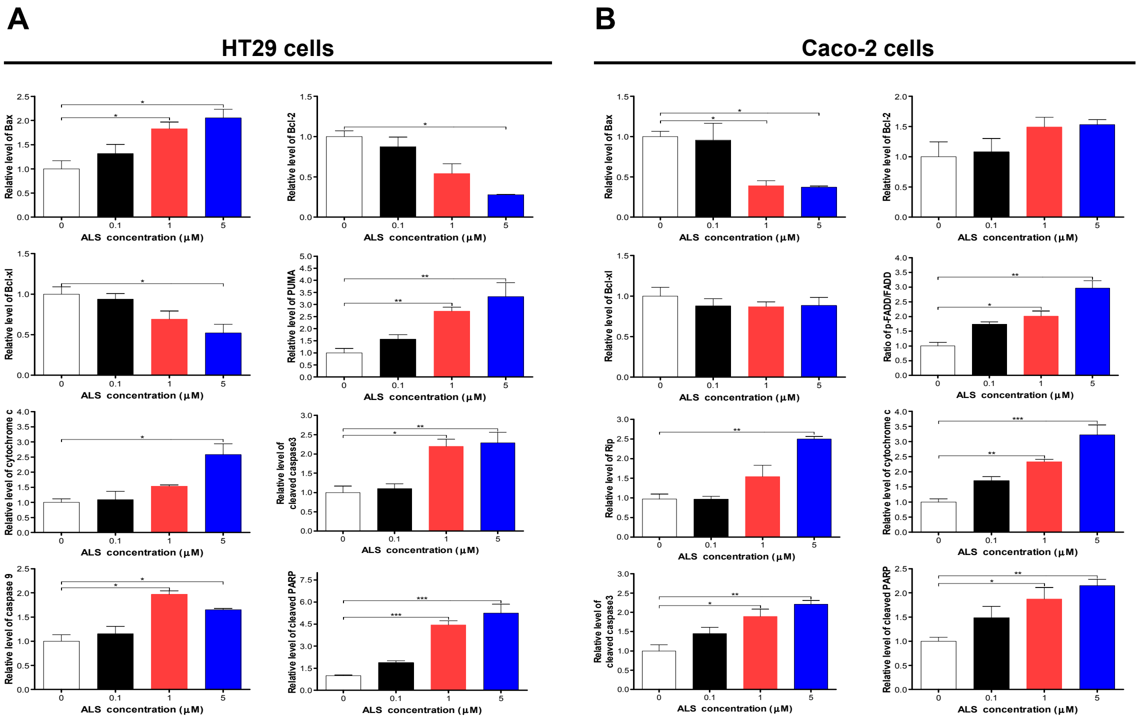

Figure S5. Effect of ALS on the expression level of key proapoptotic and antiapoptotic molecules in HT29 and Caco-2 cells. (**A**) Bar graphs showing the relative level of Bcl-xl, Bax, Bcl-2, PUMA, cytochrome c, cleaved caspase 3, cleaved caspase 9, and cleaved PARP in HT29 cells; (**B**) Bar graphs showing the relative level of Bcl-xl, Bax, Bcl-2, p-FADD (Ser194), FADD, RIP, cytochrome c, cleaved caspase 3, and cleaved PARP in Caco-2 cells. β-Actin was used as the internal control. Data are expressed as the mean SD of three independent experiments. * $p < 0.05$ and ** $p < 0.01$ by one-way ANOVA.

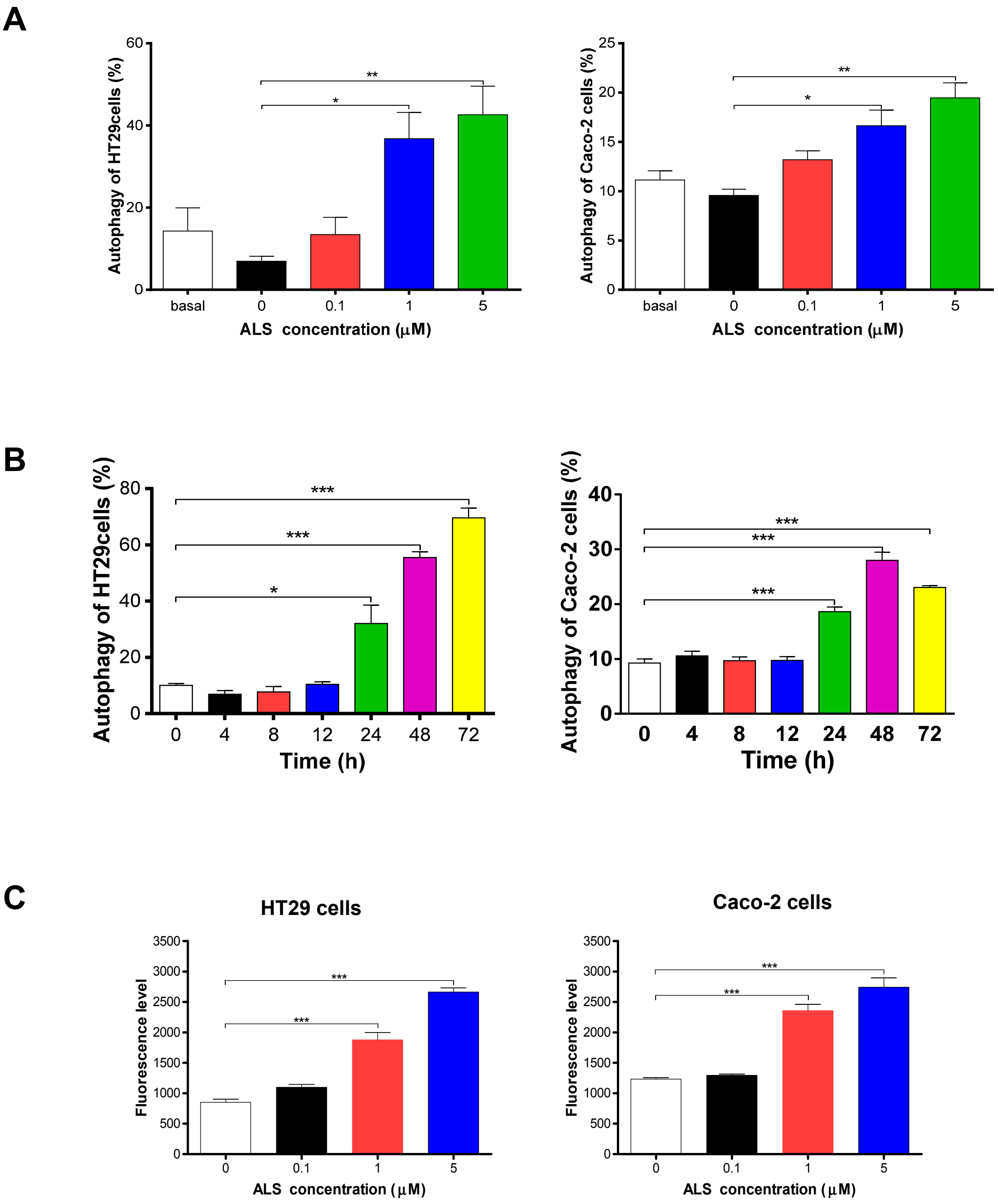

Figure S6. ALS induces autophagic cell death in HT29 and Caco-2 cells. (**A**) Bar graphs showing the percentage of autophagic HT29 and Caco-2 cells quantified by flow cytometry; (**B**) Bar graphs showing the percentage of autophagic HT29 and Caco-2 cells quantified by flow cytometry; (**C**) Bar graphs showing the intracellular autophagic level in HT29 and Caco-2 cells. Data represent the mean + SD of three independent experiments. * $p < 0.05$, ** $p < 0.01$, and *** $p < 0.001$ by one-way ANOVA.

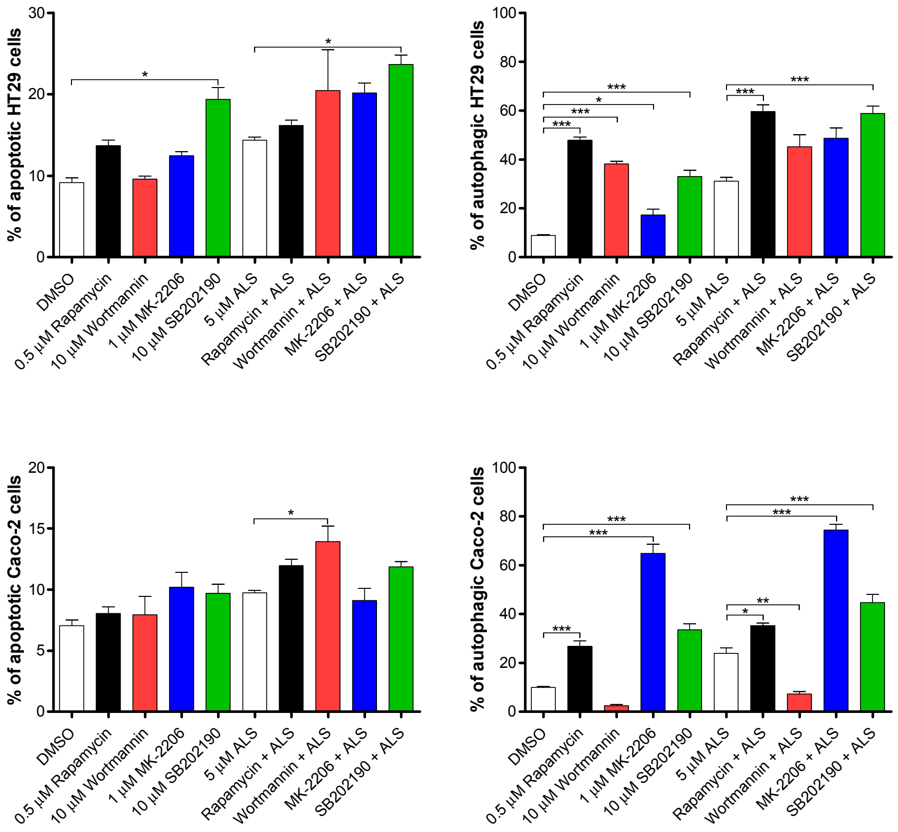

Figure S7. Effect of various inducers and inhibitors on the apoptosis and autophagy induced by ALS in HT29 and Caco-2 cells. Bar graphs showing the effect of various compounds on the apoptosis and autophagy in HT29 and Caco-2 cells. Data are shown as the mean ± SD of three independent experiments. * $p < 0.05$, ** $p < 0.01$, and *** $p < 0.001$ by one-way ANOVA.
